# Effect of Water, Sanitation, and Hygiene on the Prevention of Trachoma: A Systematic Review and Meta-Analysis

**DOI:** 10.1371/journal.pmed.1001605

**Published:** 2014-02-25

**Authors:** Meredith E. Stocks, Stephanie Ogden, Danny Haddad, David G. Addiss, Courtney McGuire, Matthew C. Freeman

**Affiliations:** 1 Department of Environmental Health, Emory University, Atlanta, Georgia, United States of America; 2 International Trachoma Initiative, Taskforce for Global Health, Decatur, Georgia, United States of America; 3 Children Without Worms, Taskforce for Global Health, Decatur, Georgia, United States of America; 4 Emory Eye Center, Emory University, Atlanta, Georgia, United States of America; University of East Anglia, United Kingdom

## Abstract

**Background:**

Trachoma is the world's leading cause of infectious blindness. The World Health Organization (WHO) has endorsed the SAFE strategy in order to eliminate blindness due to trachoma by 2020 through “surgery,” “antibiotics,” “facial cleanliness,” and “environmental improvement.” While the S and A components have been widely implemented, evidence and specific targets are lacking for the F and E components, of which water, sanitation, and hygiene (WASH) are critical elements. Data on the impact of WASH on trachoma are needed to support policy and program recommendations. Our objective was to systematically review the literature and conduct meta-analyses where possible to report the effects of WASH conditions on trachoma and identify research gaps.

**Methods and Findings:**

We systematically searched PubMed, Embase, ISI Web of Knowledge, MedCarib, Lilacs, REPIDISCA, DESASTRES, and African Index Medicus databases through October 27, 2013 with no restrictions on language or year of publication. Studies were eligible for inclusion if they reported a measure of the effect of WASH on trachoma, either active disease indicated by observed signs of trachomatous inflammation or *Chlamydia trachomatis* infection diagnosed using PCR. We identified 86 studies that reported a measure of the effect of WASH on trachoma. To evaluate study quality, we developed a set of criteria derived from the GRADE methodology. Publication bias was assessed using funnel plots. If three or more studies reported measures of effect for a comparable WASH exposure and trachoma outcome, we conducted a random-effects meta-analysis. We conducted 15 meta-analyses for specific exposure-outcome pairs. Access to sanitation was associated with lower trachoma as measured by the presence of trachomatous inflammation-follicular or trachomatous inflammation-intense (TF/TI) (odds ratio [OR] 0.85, 95% CI 0.75–0.95) and *C. trachomatis* infection (OR 0.67, 95% CI 0.55–0.78). Having a clean face was significantly associated with reduced odds of TF/TI (OR 0.42, 95% CI 0.32–0.52), as were facial cleanliness indicators lack of ocular discharge (OR 0.42, 95% CI 0.23–0.61) and lack of nasal discharge (OR 0.62, 95% CI 0.52–0.72). Facial cleanliness indicators were also associated with reduced odds of *C. trachomatis* infection: lack of ocular discharge (OR 0.40, 95% CI 0.31–0.49) and lack of nasal discharge (OR 0.56, 95% CI 0.37–0.76). Other hygiene factors found to be significantly associated with reduced TF/TI included face washing at least once daily (OR 0.76, 95% CI 0.57–0.96), face washing at least twice daily (OR 0.85, 95% CI 0.80–0.90), soap use (OR 0.76, 95% CI 0.59–0.93), towel use (OR 0.65, 95% CI 0.53–0.78), and daily bathing practices (OR 0.76, 95% CI 0.53–0.99). Living within 1 km of a water source was not found to be significantly associated with TF/TI or *C. trachomatis* infection, and the use of sanitation facilities was not found to be significantly associated with TF/TI.

**Conclusions:**

We found strong evidence to support F and E components of the SAFE strategy. Though limitations included moderate to high heterogenity, low study quality, and the lack of standard definitions, these findings support the importance of WASH in trachoma elimination strategies and the need for the development of standardized approaches to measuring WASH in trachoma control programs.

*Please see later in the article for the Editors' Summary*

## Abstract 2

Matthew Freeman and colleagues identified 86 individual studies that reported a measure of the effect of water, sanitation, and hygiene on trachoma and conducted 15 meta-analyses for specific exposure-outcome pairs.

*Please see later in the article for the Editors' Summary*

## Editors' Summary

**Background:**

Trachoma is a bacterial eye infection, which if left untreated may lead to irreversible blindness. Repeated infections over many years cause scarring on the eyelid, making the eyelashes turn inward. This causes pain and damage to the cornea at the front of the eye, which eventually leads to loss of vision. The disease is most common in rural areas in low-income countries, specifically sub-Saharan Africa. It spreads easily through contact with the discharge from an infected eye or nose, by hands, or by flies landing on the face. Women and children are more often affected than men. Trachoma is the world's leading cause of preventable blindness. A global alliance, led by The World Health Organization, is aiming to eliminate trachoma by 2020 by adopting the SAFE strategy. There are four components of this strategy. Two relate to treating the disease—“surgery” and “antibiotics.” The other two components relate to long-term prevention by promoting “facial” cleanliness and “environmental” changes (for example improving access to water and sanitation or reducing the breeding grounds for flies).

**Why Was This Study Done?:**

The SAFE approach has been very successful in reducing the number of people with trachoma from 84 million in 2003 to 21.4 million in 2012. However, it is widely recognized that efforts need to be scaled up to reach the 2020 goal. Furthermore, if current improvements are to be sustained, then more attention needs to be given to the “F” and “E” elements and effective prevention. This study aimed to identify the most effective ways to improve hygiene, sanitation, and access to water for better trachoma control, and to find better ways of monitoring progress. The overall goal was to summarize the evidence in order to devise strategic and cost-effective approaches to trachoma prevention.

**What Did the Researchers Do and Find?:**

The researchers conducted a systematic review, which involved first identifying and then assessing the quality of all of the research published on this topic. They then carried out a statistical analysis of the combined data from these studies, with the aim of drawing more robust conclusions (a meta-analysis). The analysis involved 15 different water, sanitation, and hygiene exposures (either hardware or practices, as determined by what was available in the literature) to determine which had the biggest impact on reducing the levels of trachoma. Most of the data came from studies carried out in Africa. The findings suggested that 11 of these exposures made a significant difference to the risk of infection or clinical symptoms of the disease. Improving personal hygiene had the greatest impact. Effective measures included face washing once or twice a day, using soap, using a towel, and daily bathing. Similarly, access to a sanitation facility, rather than open ground, also had a positive impact. The researchers also analyzed the data relating to water access. However, the studies so far have not yet measured this in a way that addresses the issues relevant to trachoma infection. Most studies have looked at whether the distance from a water source has an impact (and it seems it does not), whereas it may be more important to assess whether people have access to clean water or to enough water to wash. Many of these analyses require additional research to further clarify the impact of individual water, sanitation, and hygiene exposure on disease.

**What Do These Findings Mean?:**

Overall, the results support that notion that water, sanitation, and hygiene are important components of an integrated strategy to control trachoma. Based on the research available to date, the two most effective ways are face washing and having access to a household-level sanitation facility, typically a simple pit latrine. The findings also point to ways in which current policy could be improved. Firstly, public health guidance should be placing greater emphasis on keeping the face clean. Current advice tends to focus on washing with clean water, but use of soap appears more effective. There are also opportunities for organizations to collaborate in this area. For example, organizations focusing on the prevention of diarrhea in children, which promote handwashing, could at the same time campaign for face washing to reduce transmission of trachoma. The second policy area to target is access to good quality sanitation. Such policy initiatives need to be better resourced in countries where trachoma is a problem. For example, although sub-Saharan Africa has the world's highest burden of trachoma, more than 50% of households there still do not have access to any sanitation facility.

There were a number of limitations to this study, which may affect the strength of the conclusions. The researchers found that many studies on this topic were observational, meaning that they did not assess an intervention and employ a control group, thus they are of limited rigor for assessing the impact of a water, sanitation, and hygiene intervention on trachoma. There was also a lot of variation in the way that different studies had defined and measured improvements to water, sanitation, and hygiene access. This made it difficult to make comparisons. Standard methods and indicators need to be developed for this purpose. The study also highlighted gaps in the research. More work is required to determine precisely what is needed in terms of access to water to reduce the incidence of trachoma. Similarly, in terms of improving sanitation, it is still unclear whether ensuring every household has a simple, onsite facility would be more effective than providing clean communal facilities. The potential role of schools in promoting relevant public health measures also needs investigation.

**Additional Information:**

Please access these Web sites via the online version of this summary at http://dx.doi.org/10.1371/journal.pmed.1001605.

- WHO provides information on trachoma (in several languages)
- The US Centers for Disease Control and Prevention provide information on trachoma
- International Trachoma Initiative is dedicated to the goal of elimination of blinding trachoma
- The Carter Center: Trachoma Control Program has a Trachoma Health Education Materials Library
- WASHNTD has an online manual resource for NTDs for WASH policy and programming

## Introduction

Trachoma is the world's leading cause of infectious blindness, responsible for visual impairment of an estimated 2.2 million people, of whom 1.2 million are irreversibly blind [1],[2]. Repeated infection with the bacteria *C. trachomatis* results in scarring of the conjunctiva of the upper eyelid that inverts the eyelids (entropion) leading to eyelashes touching the cornea and conjunctiva (trichiasis). Abrasions and secondary infections of the cornea then lead, without preventive surgery, to irreversible blindness [3],[4]. Although great progress has been made, the disease remains endemic in 53 countries, primarily in sub-Saharan Africa, the Middle East, and Asia [2]. Approximately 334,000 disability-adjusted life years are currently lost due to trachoma infection [5]. An estimated 229 million people live in endemic areas, including 176 million in Africa, though 80% of the global burden is now limited to 14 countries [6].

Intensive effort since the founding of the Global Alliance for Elimination of Blinding Trachoma by 2020 (GET 2020) has led to considerable reduction in the global burden of trachoma from 84 million cases of active trachoma in 2003 to 21.4 million in 2012 [7]–[10]. Success of trachoma control has been in part due to the World Health Organization (WHO)-endorsed SAFE strategy: a simple, low-cost “surgery” for patients with advanced stages of the disease, treatment with the “antibiotics” azithromycin or tetracycline eye ointment, promotion of “facial” cleanliness, and “environmental” improvement, which encompasses promotion of sanitation construction and increased water access [9].

The World Health Assembly (WHA) in resolution 51.11 has called for the elimination of blinding trachoma by 2020 [8],[9]. Donation of Zithromax (azithromycin) by Pfizer Inc. has enabled scale-up of efforts to reduce disease burden in relation to these targets. Annual treatments have increased from 1 million doses in 1998 to 47.8 million doses in 2012 [2] and more than 30 countries have articulated national trachoma strategies and undertaken trachoma elimination programs [11]. However, these efforts alone will not lead to sustainable elimination of blinding trachoma, and it is widely recognized that scale-up of the full SAFE strategy is needed to reach 2020 targets [12]. In particular, improvements in environmental conditions, most notably hygiene and sanitation, are needed for sustained reductions in disease burden, and no single tool can be recommended for the F and E components of SAFE [13],[14]. As pointed out by the editors of the Lancet in 2012 in response to the launch of *Accelerating Work to Overcome the Global Impact of Neglected Tropical Diseases* and reiterated by key stakeholders in the trachoma control community [15], the inclusion of water, sanitation, and hygiene as a strategic goal within the global strategy did not include specific goals or targets [16].

There is a need for increased intersectoral collaboration between neglected tropical disease (NTD) and water, sanitation, and hygiene (WASH) sectors, as well as for increased financial resources for WASH interventions in the context of trachoma control [17]. However, there is little evidence to support specific WASH interventions within the SAFE framework. While there have been successes in integrating WASH into trachoma control [15], a need remains for empirical guidance on the impact of the F and E components and for guidance on how to monitor progress. Recent Cochrane reviews on the environmental improvement (sanitation) component [18] and on face washing [19] revealed few rigorous randomized trials. The most recent comprehensive reviews of the impact of sanitation access and face washing on trachoma were conducted in 2000 [13],[14]. We found no previous reviews of all facets of WASH on trachoma.

We conducted a systematic review and meta-analysis to quantify the relationship between WASH exposures and *C. trachomatis* infection and active disease. Our purpose was to assess and present the available evidence in order to inform policy for effective and cost-effective integration of WASH for trachoma control, as well as to inform WASH monitoring indicators for trachoma control programs.

## Methods

### Search Strategy

We performed a systematic review and meta-analysis of the literature to address the effects of WASH exposures on infection and clinical signs of trachoma. We systematically searched PubMed, Embase, ISI Web of Knowledge, MedCarib, Lilacs, REPIDISCA, DESASTRES, and African Index Medicus databases with no restrictions on language or year of publication. Our search was performed through October 27, 2013 with no restriction of start date. We employed a broad set of search terms, pairing the term [trachom*] with the following WASH-related keywords: [clean* fac*], [environment*], [excre*], [face washing], [facewashing], [faec*], [fec*], [hand washing], [handwashing], [hygiene], [latrine*], [sanitation], [toilet*], [towel*], [wash cloth*], [waste], and [water]. In addition, we examined seven previous reviews pertaining to trachoma and some aspect of WASH [3],[14],[18]–[22], and hand searched the bibliographies of all relevant publications. Any additional articles found to be pertinent during this process were included.

Studies were included in the systematic review only if they measured WASH exposure, trachoma infection, and attempted to quantify the association between a WASH exposure, condition, or risk factor on trachoma. All study types were eligible if they met these inclusion criteria, and we employed no additional exclusion criteria in our approach. If an article was considered relevant, but data were not available in the format needed for our meta-analysis, the corresponding authors were contacted by e-mail and asked to supply the relevant data. We conducted meta-analyses for specific exposure-outcome relationships based on available data. Meta-analyses were conducted in adherence to the PRISMA statement (Text S1) and the MOOSE guidelines for reporting meta-analyses of observational studies [23]. Our complete protocol is available in Text S2.

### Selection Criteria and Data Extraction

Articles were selected for inclusion using a two-step review process. First, the titles and abstracts of all identified studies were examined, and studies that failed to meet the inclusion criteria after this step were excluded. Second, two reviewers (MES and CM) independently examined the full text of potentially relevant articles using a standard protocol developed by MES and MCF. In the event of disagreement regarding the eligibility of a study during this phase, the opinion of a third reviewer (MCF) was sought, and the parameters of the study's inclusion were discussed until consensus was reached.

Once a set of eligible studies was agreed upon, relevant data were extracted from each study by MES using a standard protocol. To ensure extraction reliability, CM also extracted data from a subset of 10% of identified studies, and no discrepancies were found. Data extracted included a brief description of the study (e.g., study design, setting, year, and sample size), details of the study population, and measures of trachoma. Measures of trachoma included *C. trachomatis* infection assessed by laboratory analysis of ocular swabs, most commonly using PCR or ELISA techniques, and clinical signs of active trachoma. Clinical signs of trachoma were diagnosed by trained observers using torchlights and 2.5× magnifying loupes [24]. In this approach, eyes are graded according to the WHO simplified grading system and assigned one or more of the following grades: trachomatous inflammation-follicular (TF), trachomatous inflammation-intense (TI), trachomatous scarring (TS), trachomatous trichiasis (TT), and corneal opacity (CO) [25]. Detailed descriptions of all WASH-related conditions, risk factors, or interventions were assessed and coded on the basis of the descriptions in Table 1. Primary WASH components were defined on the basis of the data available from the review.

**Table 1:** Summary of literature density results for overall systematic review.

WASH Exposure	Article Count
**Water**	**56**
Distance to water^a^	36
Type of water source	19
Access to water	7
Quantity used for washing	6
Total quantity of water	5
**Sanitation**	**51**
Sanitation access^a^	38
Sanitation use^a^	8
Sanitation type	6
Sanitation maintenance	3
Sanitation education	3
**Hygiene**	**62**
Facial cleanliness^a^	33
Face-washing frequency^a^	19
Ocular discharge^a^	14
Nasal discharge^a^	13
Soap use^a^	7
Hygiene education	6
Towel use^a^	6
Bathing frequency^a^	5
Nose wiping practices	5
Towel sharing	3

a Exposures with sufficient number of comparable measures of effect to warrant a meta-analysis.

### Quality Issues

In order to determine the quality of identified studies, we developed a set of criteria derived from the GRADE methodology [26]. Our criteria took into account diagnostic features, assessment of WASH-related risk factors, study design, and overall strengths and limitations of the studies. Studies could obtain an overall score ranging between −1 and +6 points for each meta-analysis. Studies that employed a rigorous diagnostic approach (e.g., PCR or other laboratory technique used to assess infection status) received +1 point, while studies that relied only upon clinical diagnosis of trachoma through eye examinations were given 0 points. A study was given +1 point if WASH conditions were investigated directly by the research team (e.g., directly observed the presence of sanitation facility or distance to water source). However, no point was assigned if this was assessed using only a questionnaire. Studies with quasi-experimental or experimental designs were awarded +1 point, while observational studies were awarded 0 points. Studies that purposively calculated sample size to address trachoma as a key outcome of WASH characteristics were awarded +1 point. Studies that attempted to control for confounding factors were assigned an additional +1 point. A list of the confounders each study attempted to control for is available in Text S3. Other strengths and limitations of each study were assessed, and the study was assigned +1 point for additional strengths, and −1 points for additional limitations. MES performed the quality assessment independently and documented the results in separate tables. All relevant studies were included in the review regardless of their overall quality rating. Quality ratings did not affect the meta-analysis or subsequent summary of effect measures, but help to demonstrate the overall quality of individual studies and to identify research gaps. The worksheet used in grading studies for each meta-analysis is available in Text S4.

### Meta-Analysis

We conducted meta-analyses for all WASH-related exposures for which three or more studies reported comparable odds ratios (ORs) for the same WASH–trachoma association (i.e., ORs describing the effect of similarly defined exposures on the same measure of trachoma). All ORs used in meta-analyses reflected the results of cross-sectional risk factor analyses. WASH conditions included sanitation access, sanitation use, distance to water of less than 1 km, clean face, lack of ocular discharge, lack of nasal discharge, washing face at least once daily, washing face at least twice daily, bathing at least once daily, towel use, and soap use. Data were stratified by clinically relevant trachoma measures: *C. trachomatis* infection and the most commonly reported measure of active trachoma, trachomatous inflammation-follicular and/or trachomatous inflammation-intense (TF/TI). Reported ORs served as effect measures. Where a study reported more than one OR for the same WASH exposure (i.e., one for the entire population and one for a sub-population), we chose for inclusion the OR most similar to others in the meta-analysis. When both unadjusted and adjusted ORs were reported, adjusted ORs were included in meta-analysis [27], and adjusted ORs are indicated with asterisks within the forest plots. When ORs were not reported, they were calculated from 2×2 contingency tables; ORs that were calculated using data provided by contacted authors are indicated as such by a footnote in the forest plot. Findings of those studies identified in the systematic review, but not included in meta-analyses were summarized and examined for patterns within and between WASH subcomponent measures, as recommended by the Cochrane Collaboration [27].

We used Microsoft Excel (Microsoft Corporation) to conduct meta-analyses and to develop forest plots [28]. Funnel plots were utilized to investigate the existence of publication bias [29]. Heterogeneity between studies was determined using Higgins' *I* ^2^ and Cochran's Q-tests [27]. When heterogeneity was moderate to high (*I^2^*>50%), sub-group analyses were performed to identify potential sources of heterogeneity. Random effects models were used throughout to enhance generalizability of results [30], and pooled ORs for the effect of the selected WASH conditions on trachoma were employed [27]. Associations between WASH conditions and trachoma were reoriented so that all ORs reflect the relationship between *improved* WASH conditions on trachoma (e.g., all facial cleanliness ORs were converted to reflect the effect of having a clean face, rather than dirty face, on odds of trachoma).

## Results

### Characteristics of Identified Studies

**Figure 1:**
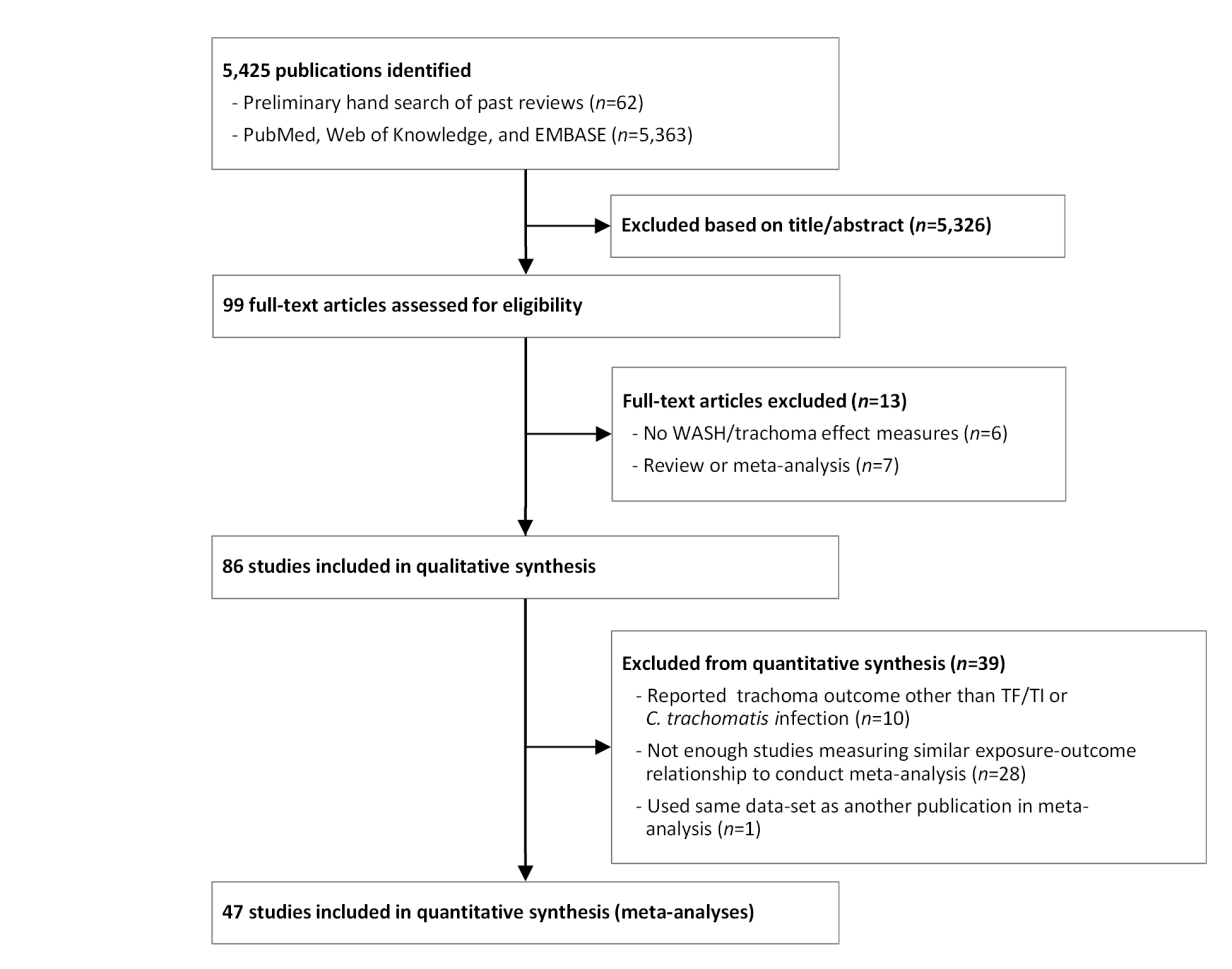
Flow chart of publications identified and excluded for this review.

**Figure 2:**
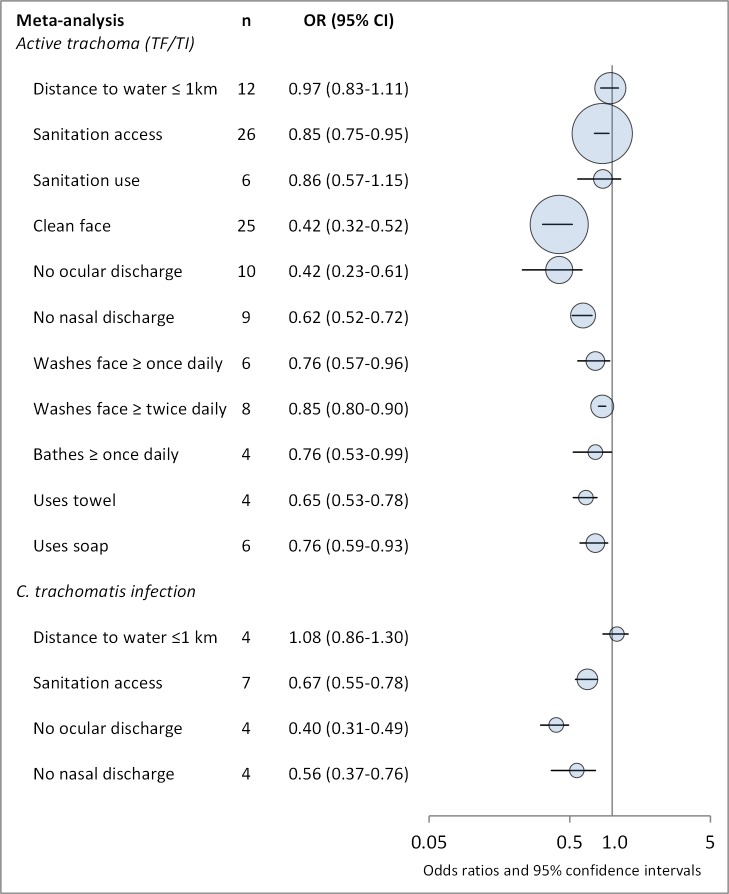
Summary of meta-analyses examining association of WASH exposures with active trachoma (TF/TI) and *C. trachomatis* infection. Circles indicate ORs, while the size of circles represents the number of studies included in the meta-analysis (*n*). Horizontal lines represent 95% CIs.

Our initial search yielded 5,425 publications (Figure 1). Ninety-nine publications were deemed relevant after review of titles and, when available, abstracts. These articles were fully screened by MES and CM. Following this screening, 86 articles were determined to meet systematic review inclusion criteria (Tables 2–8). We conducted a total of 15 meta-analyses. A summary of the calculated pooled ORs and 95% CIs is provided in Figure 2 and Table 9. Forty-six studies were included in at least one meta-analysis, and 33 appeared in two or more meta-analyses.

**Table 2:** Summary of publications reporting only on water-related risk factors.

Reference	Study Design and Setting	Year	Study Population	WASH Components	Type of Trachoma Measure	Diagnostic Approach	Data Obtained	Assessment Method		
								W	S	H
Alemu et al. [48]	Intervention study in Dalocha District, Central Ethiopia	1998–2002	644 children between aged 1–9 years	Access to water	TF/TI	Clinical exam	Change in prevalence	Q	—	—
Assaad et al. [49]	Descriptive study on Island of Taiwan	1960–1961	Pre-WWII Chinese immigrants in sample units of approximately 125 people	Distance to water, type of water source	TF/TI	Clinical exam	Age-standardized trachoma rate	Q, O	—	—
Cairncross et al. [50]	Descriptive study in two villages in the Mueda Plateau in Northern Mozambique	1983	100 households in each village	Water access	Any trachoma sign	Clinical exam	X^2^	O	—	—
Caligaris et al. [51]	Descriptive study in nine day care centers of Bela Vista district in Sao Paulo, Brazil	1995	All preschool students attending day care centers, aged 0–7 years	Water access	TF/TI, *C. trachomatis* infection	DFA	OR	Q	—	—
Chumbley et al. [52]	Descriptive study in West Bank and Gaza Strip	1982– 1983	9,058 Palestinian Arabs	Water access	Any trachoma sign	Clinical exam	2×2 (from bar chart)	O	—	—
Hoeschmann et al. [53]	Descriptive study in Malawi	1999	1,363 adults >50 years, 2,251 children aged 1–6 years	Distance to water	TF, TI, TF/TI	Clinical exam	OR	Q	—	—
Marshall et al. [54]	Descriptive study in Naha, Japan	1962	58,480 primary and junior high students	Type of water source	TF/TI	Clinical exam	Change in prevalence	O	—	—
Polack et al. [55]	Descriptive study in sub-village of Kahe Mpya in Rombo District, northern Tanzania	2000	All consenting residents (*n* = 956)	Distance to water	TF/TI, *C. trachomatis* infection	Clinical exam, PCR	OR	Q, O	—	—
West et al. [56]	Descriptive study in 20 villages in Kongwa sub-district, Dodoma region, Tanzania	n.s.	All preschool-age children and caretakers in randomly selected sub-districts	Water access	TF/TI	Clinical exam	Prevalence ratio	O	—	—

H, hygiene; O, observed directly; Q, questionnaire; S, sanitation; W, water.

**Table 3:** Summary of publications reporting only on sanitation-related risk factors.

Reference	Study Design and Setting	Year	Study Population	WASH Components	Type of Trachoma Measure	Diagnostic Approach	Data Obtained	Assessment Method		
								W	S	H
Courtright et al. [57]	Descriptive study in the hamlet of Kafr in the Egyptian Nile Delta	1987	225 children aged 1–5 years from 96 households	Sanitation presence	TI	Clinical exam	OR	—	Q, O	—
Emerson et al. [58]	Intervention study in rural communities in the North Bank and Central River divisions of The Gambia	1999–2001	All residents over 4 months of age (*n* = 6,087)	Sanitation presence	TF/TI	Clinical exam	Change in prevalence	—	O	—
Haile et al. [59]	Descriptive study following an antibiotic and sanitation promotion intervention in twelve communities in Amhara region of Ethiopia.	2006–2008	50 randomly selected sentinel children aged 0–9 years from each of the 12 communities.	Sanitation use	TF/TI, *C. trachomatis* infection	Clinical exam, PCR	Difference in prevalence	—	O	—
Montgomery et al. [60]	Descriptive study in seven rural Tanzanian communities	2007	594 households (92 cases, 502 controls)	Sanitation type, sanitation use	TF/TI	Clinical exam	OR	—	Q O	—
Reilly et al. [61]	Descriptive study in Marajo Island in Para State, Brazil	2004	180 adults (>15 years) and 187 children (0–15 years)	Sanitation presence	Any trachoma sign	Clinical exam	Regression coefficient	—	Q, O	—
Stoller et al. [37]	Intervention study in 24 communities in Amhara state, Ethiopia	2006–2008	60 randomly selected children aged 0–9 years from each sentinel site	Sanitation presence	TF/TI, *C. trachomatis* infection	Clinical exam, PCR	Change in prevalence	—	O	—

H, hygiene; O, observed directly; Q, questionnaire; S, sanitation; W, water.

**Table 4:** Summary of publications reporting only on hygiene-related risk factors.

Reference	Study Design and Setting	Year	Study Population	WASH Components	Type of Trachoma Measure	Diagnostic Approach	Data Obtained	Assessment Method		
								W	S	H
Alene et al. [62]	Descriptive study in Jangua Mariam, North Western Ethiopia	1998	1,718 individuals of all age groups in 414 households	Face-washing frequency	Any trachoma sign	Clinical exam	OR	—	—	Q
De Sole et al. [29]	Intervention study in a primary school in Metahara, Ethiopia	1984–1985	239 children aged <10 years at primary school	Hygiene education	TF/TI	Clinical exam	Change in prevalence	—	—	O
Gower et al. [63]	Descriptive study in Maindi and Kahe Mpya, Tanzania and a cluster of 14 small villages in Jareng, Upper Saloum District, The Gambia	n.s.	1,128 children aged <9 years	Nasal discharge	TF, TF/TI	Clinical exam	2×2	—	—	O
Guraksin et al. [64]	Descriptive study in Koprukoy and Pasinler districts in Turkey	1993–1994	6,386 individuals aged <30 years, 2,220 individuals aged >30 years	Face-washing frequency, soap use, towel sharing	TF/TI	Clinical exam	X^2^	—	—	Q
Hagi et al. [65]	Descriptive study in 203 villages in Mali	1996–1997	14,627 children aged <10 years in 2,269 randomly selected households	Facial cleanliness, flies on face, towel use	TF/TI	Clinical exam	OR	—	—	Q, O
Hall et al. [66]	Descriptive study in Ethiopia	n.s.	7,572 school-children aged 7–17 years	Face-washing frequency	Any trachoma sign	Clinical exam	OR	—	—	Q
Khanduja et al. [67]	Descriptive study in10 rural villages in Haryana, India	2006	1,000 children aged 1–9 years	Facial cleanliness	TF/TI, *C. trachomatis* infection	Clinical exam, PCR	OR	—	—	O
King et al. [68]	Descriptive study in one Malian and two Tanzanian villages	2006	All 424 children aged 1–5 years	Ocular discharge	TF/TI	Clinical exam	OR	—	—	O
Koizumi et al. [69]	Descriptive study in 10 Regional Health Administration Areas (RHAA) in Sao Paulo, Brazil	1999	27,091 public preschool and school children aged 4–14 years	Facial cleanliness	Any trachoma sign	Clinical exam	2×2	—	—	O
Roba et al. [70]	Descriptive study in Enemor and Ener District of Curage Zone, Ethiopia	2006	848 children aged 1–9 years	Facial cleanliness	TF/TI	Clinical exam	OR	—	—	O
West et al. [71]	Descriptive study in 2 villages in Kongwa, Tanzania	n.s.	500 children aged 1–7 years	Facial cleanliness, ocular discharge, nasal discharge, flies on face	TF/TI	Clinical exam	OR	—	—	O
West et al. [38]	Intervention study in 3 pairs of villages in the Kongwa subdistrict of central Tanzania	1989–1990	1,417 children aged 1–7 years enrolled, 1,168 participated in all four examinations.	Facial cleanliness, hygiene education	Any trachoma sign, TI	Clinical exam	OR	—	—	O
Wilson et al. [72]	Descriptive study in 5 villages in Chiapas, Mexico	n.s.	475 children in villages aged 2–10 years, primarily from farming families of Mayan descent	Nose wiping practices	TI	Clinical exam	OR	—	—	Q

H, hygiene; O, observed directly; Q, questionnaire; S, sanitation; W, water.

**Table 5:** Summary of publications reporting on water- *and* sanitation-related risk factors.

Reference	Study Design and Setting	Year	Study Population	WASH Components	Type of Trachoma Measure	Diagnostic Approach	Data Obtained	Assessment Method		
								W	S	H
Ezz al Arab et al. [73]	Descriptive study in Menofiya governate in Nile Delta, Egypt	n.s.	3,272 children aged 2–6 years and 3,322 adults aged >50 years	Type of water source, sanitation type	TF/TI	Clinical exam	X^2^	Q	Q	—
Katz et al. [74]	Descriptive study in Sarlahi district, Nepal	1990–1991	836 children aged 24–76 months	Type of water source, water access, sanitation access	TF/TI	Clinical exam	OR	Q	—	—
Luna et al. [75]	Descriptive study in Bebedouro municipality in Sao Paulo	1986	1,959 children aged 1–10 years in 1,416 households.	Type of water source, total quantity of water, sanitation presence, sanitation type	TF/TI	Clinical exam	OR, 2×2	O	n.s.	—
Montgomery et al. [76]	Descriptive case-control study in eight villages in Kongwa district, Tanzania	2006–2008	678 households (95 cases, 583 controls) with a sentinel child aged 1–5 years	Distance to water, sanitation use, sanitation maintenance	TF/TI	Clinical exam	OR, 2×2	Q	Q, O	—
Ngondi et al. [77]	Descriptive study in 11 districts in Southern Sudan	2001–2006	11,155 children aged 1–14 years from 3,950 households	Distance to water, type of water source, sanitation presence	TT	Clinical exam	OR	Q	Q, O	—
Sallam et al. [78]	Descriptive study in Yemen	2001	787 randomly selected school children under age 20	Type of water source, sanitation presence	*C. trachomatis* infection	PCR	OR	Q	Q	—
Zerihun et al. [79]	Descriptive study in 14 villages and 1 town in Dedo, Manna, Sekka, and Kossa districts of Jimma Zone, Ethiopia	1994–1995	7,423 residents of 100 randomly selected households in each village	Distance to water, presence of sanitation	TF/TI, TS	Clinical exam	OR	Q	Q	—

H, hygiene; O, observed directly; Q, questionnaire; S, sanitation; W, water.

**Table 6:** Summary of publications reporting on water- *and* hygiene-related risk factors.

Reference	Study Design and Setting	Year	Study Population	WASH Components	Type of Trachoma Measure	Diagnostic Approach	Data Obtained	Assessment Method		
								W	S	H
Baggaley et al. [80]	Descriptive study in randomly selected households in 64 villages in Rombo district, Tanzania	2002	12,415 children aged 1–9 years	Distance to water, flies on face	TF/TI	Clinical exam	OR	Q, O	—	O
D'Amaral et al. [81]	Descriptive case-control study in Greater Metropolitan Sao Paulo, Brazil	1999	121 pairs of cases/controls matched by age and school	Water access, face-washing frequency	TF/TI	Clinical exam	OR	Q	—	Q
Hsieh et al. [82]	Descriptive study in Kongwa, Tanzania	1989–1995	176 children who were 1–2 years old in 1989 survey and available for follow-up in 1995	Distance to water, facial cleanliness	TF/TI	Clinical exam	OR	Q	—	O
Schemann et al. [83]	Descriptive study in 210 villages in Mali	n.s.	All children aged <10 years and women aged >14 years in randomly chosen households	Distance to water, facial cleanliness, flies on face	TF/TI, TS, TT, CO	Clinical exam	OR	Q	—	O
Taylor et al. [84]	Descriptive study in 2 communities in Chiapas, Mexico	n.s.	Entire population of both communities (*n* = 1,097)	Face-washing frequency, nose wiping practices	Any trachoma sign	Clinical exam	Relative risk, X^2^	—	—	Q
West et al. [85]	Descriptive study in 20 villages in the Kongwa, Tanzania	1986	3,800 children aged 1–7 years	Distance to water, total quantity of water, facial cleanliness	TF/TI	Clinical exam	OR	Q	—	O
West et al. [86]	Descriptive study in 9 villages in Tanzania	n.s.	1,085 children aged 1–7 years	Distance to water, facial cleanliness, nose wiping practices	TF/TI, *C. trachomatis* infection	Clinical exam, PCR	OR	Q	—	O
West et al. [87]	Intervention study in 3 pairs of villages in the Kongwa subdistrict of central Tanzania	1989–1990	1,417 children aged 1–7 years enrolled, 1,168 participated in all four examinations	Distance to water, hygiene education, facial cleanliness	TI	Clinical exam	OR, 2×2	Q	—	O

H, hygiene; O, observed directly; Q, questionnaire; S, sanitation; W, water.

**Table 7:** Summary of publications reporting only on sanitation- *and* hygiene-related risk factors.

Reference	Study Design and Setting	Year	Study Population	WASH Components	Type of Trachoma Measure	Diagnostic Approach	Data Obtained	Assessment Method		
								W	S	H
Abdou et al. [88]	Intervention study in 10 randomly selected communities in Maradi, Niger	2005–2008	557 randomly selected sentinel children aged 1–5 years from each village	Hygiene education, sanitation education	TF/TI, *C. trachomatis* infection	Clinical exam, PCR	OR	—	O	O
Burton et al. [89]	Descriptive study in Upper Saloum District, The Gambia	n.s.	All available residents (*n* = 1,319)	Sanitation presence, ocular discharge, nasal discharge, flies on face	*C. trachomatis* infection	PCR	OR	—	Q	O
Burton et al. [90]	Descriptive study in 14 villages in rural Gambia	2001–2003	All consenting residents living in the area for more than 6 months (*n* = 1,319)	Sanitation presence, ocular discharge, nasal discharge, flies on face	*C. trachomatis* infection	PCR	OR	—	Q	O
Hassan et al. [91]	Descriptive study in 88 districts in 12 northern states of Sudan	2006– 2010	Children aged 1–9 years	Sanitation access, face-washing frequency	TF/TI	Clinical exam	OR	—	O	Q
Khandekar et al. [92]	Intervention study in villages My Thon and Xom Ngoai, Vietnam	2002–2005	All households and residents of all ages comprised target population	Sanitation education, hygiene education	TF/TI	Clinical exam	Additional decline in prevalence due to FE	—	O	O
Resnikoff et al. [93]	Intervention study in four villages in Oulessebougou, Mali	1994	347 subjects with TF/TI	Sanitation education, hygiene education	TF/TI	Clinical exam	OR	—	O	O
Rubinstein et al. [94]	Intervention study in four villages in the Egyptian delta	1991–1992	Children aged <10 years	Sanitation use, sanitation education, nasal discharge, flies on face, hygiene education, soap use	Any trachoma sign	Clinical exam	Relative risk	—	Q, O	Q, O
Schemann et al. [95]	Descriptive study in Burkina Faso	1997	All children aged <10 years in randomly selected villages (*n* = 16,514)	Sanitation presence, face-washing frequency, bathing frequency, facial cleanliness, flies on face	TF/TI, TI	Clinical exam	OR	—	O	Q, O
Vinke et al. [96]	Descriptive study in Kembata Tembaro Zone in Ethiopia	n.s.	1,513 children aged 1–9 years from 306 households	Distance to sanitation, facial cleanliness	TF/TI	Clinical exam	OR	—	Q, O	O

H, hygiene; O, observed directly; Q, questionnaire; S, sanitation; W, water.

**Table 8:** Summary of publications reporting on water-, sanitation-, *and* hygiene-related risk factors.

Reference	Study Design and Setting	Year	Study Population	WASH Components	Type of Trachoma Measure	Diagnostic Approach	Data Obtained	Assessment Method		
								W	S	H
Abdou et al. [97]	Descriptive study in 12 randomly selected villages in Niger	n.s.	641 children aged 1–5 years	Distance to water, sanitation presence, facial cleanliness, flies on face	TF/TI, *C. trachomatis* infection	Clinical exam, PCR	OR	Q	O	O
Amza et al. [98]	Descriptive study in 48 randomly selected Nigerian communities	2010	24,536 participants in study, including 4,484 sentinel children aged 0–5 years	Distance to water, sanitation presence, ocular discharge, nasal discharge, flies on face	TF/TI, *C. trachomatis* infection	Clinical exam, PCR	Regression coefficient	Q	Q	O
Ayele et al. [99]	Descriptive study in 12 communities in Goncha Siso Enese woreda, Amhara Region, Ethiopia	2009	50 children aged 0–9 years from each of 12 communities (*n* = 575)	Distance to water, sanitation presence, ocular discharge, nasal discharge, flies on face	TF/TI, *C. trachomatis* infection	Clinical exam, PCR (DNA and RNA)	OR	Q	Q	O
Bailey et al. [100]	Descriptive case-control study in village of Keneba in the Kiang West district of The Gambia	1987	Cases: 18 mothers whose children had one or more cases of active trachoma (*n* = 68, 33 with TF/TI). Controls: 16 mothers with trachoma-free children (*n* = 50)	Quantity of water used for washing, sanitation presence, facial cleanliness, face-washing frequency, bathing frequency, soap use, towel sharing	Any trachoma sign	Clinical exam	OR	O	O	Q, O
Cajas-Monson et al. [101]	Descriptive study in 4 villages in Kongwa district, Tanzania	2009	1,991 children aged <9 years	Time to water, sanitation presence, facial cleanliness	TF/TI, *C. trachomatis* infection	Clinical exam, PCR	2×2	Q	O	O
Cruz et al. [35]	Descriptive study in San Gabriel da Cachoeira, Brazil	n.s.	440 children aged <9 years, 1,069 adults aged >15 years	Type of water source, sanitation type, sanitation use, facial cleanliness, ocular discharge, towel use	Any trachoma sign	Clinical exam	Prevalence ratio	Q, O	Q, O	Q, O
Cumberland et al. [102]	Descriptive study in Gurage, Oromia, and South Welo zones of Ethiopia	2002	1,960 children aged 3–9 years in selected households	Type of water source, distance to water, sanitation use, bathing frequency, ocular discharge, nasal discharge, flies on face	TF/TI	Clinical exam	OR	Q, O	Q, O	Q, O
Edwards et al. [103]	Descriptive study in 40 randomly selected villages in Unity State, South Sudan	2002–2005	1,722 children aged 3–9 years	Distance to water, type of water source, sanitation presence, sanitation use, face-washing frequency, ocular discharge, flies on face, towel sharing	TF/TI	Clinical exam	OR	Q	Q, O	Q, O
Edwards et al. [104]	Descriptive study in 37 Ethiopian communities	2010	All consenting residents (*n* = 5,727)	Distance to water, type of water source, sanitation presence, ocular discharge, nasal discharge	TF, TT	Clinical exam	OR	Q	Q	O
Ejigu et al. [105]	Descriptive study in Kersa District, Southwest Ethiopia	2011	305 children aged 1–9 years	Distance to water, sanitation presence, facial cleanliness	TF/TI	Clinical exam	OR	Q	O	O
Faye et al. [106]	Descriptive study in Nioro department, Kaolack Region, Senegal	2003	1,648 randomly selected children aged 2–5 years	Distance to water, quantity of water used for washing, sanitation presence, sanitation use, facial cleanliness, flies on face, face-washing frequency, soap use	TF/TI	Clinical exam	OR	Q	Q	Q, O
Golovaty et al. [107]	Descriptive cross-sectional study in one community in Ankober, Ethiopia	2007	507 children aged 1–9 years	Type of water source, sanitation presence, facial cleanliness, flies on eyes	TF/TI	Clinical exam	OR	Q	Q	O
Harding-Esch et al. [103],[108]	Descriptive study in the Gambia's Lower River Region and North Bank Region	n.s.	Children aged <10 years	Distance to water, type of water source, sanitation presence, sanitation type, facial cleanliness, ocular discharge, nasal discharge	TF/TI	Clinical exam	OR	Q	Q	O
Harding-Esch et al. [109]	Descriptive study in 48 Gambian and 36 Tanzanian communities	n.s.	5,036 randomly selected children aged 0–5 years	Distance to water, sanitation presence, ocular discharge, nasal discharge, flies on face, hygiene education	*C. trachomatis* infection	PCR	OR	Q	Q	O
Jip et al. [110]	Descriptive study in 10 local government areas in Katsina State, Nigeria	n.s.	11,407 children and 8,901 adults from 2,244 households	Distance to water, water access, sanitation presence, facial cleanliness, face-washing frequency, ocular discharge, nasal discharge, flies on face,	TF/TI	Clinical exam	OR	Q	Q	Q, O
Kalua et al. [36]	Descriptive study in Chikwawa and Mchinji districts, Malawi	n.s.	Children aged 1–9 years (*n* = 1,135 in Chikwawa, *n* = 1,295 in Mchinji)	Water access, type of water source, sanitation presence, sanitation type, facial cleanliness	TF	Clinical exam	OR	Q	O	O
Ketema et al. [111]	Descriptive study in 5 randomly selected villages in Baso Liben District, East Gojjam, Amhara Regional state, Ethiopia	2012	792 children aged 1–9 years	Distance to water, total quantity of water, sanitation use, facial cleanliness, soap use	TF/TI	Clinical exam	OR	Q	Q	Q, O
Khandekar et al. [112]	Descriptive study in Nizwa, Oman	2002	229 children aged 0–15 years	Water source type, sanitation maintenance, facial cleanliness	TF/TI	Clinical exam	2×2	Q	O	O
Lucena et al. [113]	Descriptive study in Brazil	2007	412 individuals aged 1–86 years	Type of water source, sanitation presence, facial cleanliness	Any trachoma sign	Clinical exam	2×2	Q	Q	Q, O
Mahande et al. [114]	Descriptive study in Maasai village, Hai district, Tanzania	2005	Households with two or more children aged 1–9 years	Distance to water, quantity of water used for washing, total quantity of water, sanitation presence, face-washing frequency, sharing water for washing, towel sharing, soap use	TF/TI	Clinical exam	OR	Q	Q	Q
Mesfin et al. [115]	Descriptive study in 48 villages in Tigray, Ethiopia	n.s.	3,900 randomly selected people from 1200 households	Distance to water, sanitation presence, face-washing frequency, soap use	TF/TI	Clinical exam	OR	Q, O	Q, O	Q
Mpyet et al. [116]	Descriptive study in 27 villages in Yobe state, Nigeria	n.s.	639 children aged 1–5 years	Distance to water, quantity of water used for washing, sanitation presence, facial cleanliness, flies on face	TF/TI	Clinical exam	OR	Q	O	O
Mpyet et al. [117]	Descriptive population-based cross-sectional survey using multistage cluster random sampling in Kano state, Nigeria	2008	4,491 people, including 1,572 aged <10 years	Quantity of water used for washing, sanitation presence, facial cleanliness, flies on face	TF/TI	Clinical exam	OR	Q	Q	O
Ngondi et al. [118]	Descriptive study in 10 sites in southern Sudan	2001– 2005	7,418 children aged 1–9 years	Distance to water, sanitation presence, facial cleanliness, face-washing frequency	TF/TI	Clinical exam	OR	Q	Q, O	Q, O
Ngondi et al. [119]	Descriptive study in Amhara Regional State, Ethiopia	2006–2007	5,427 children aged 1–9 years from 2,845 households and 9,098 adults aged 15 and up from 4,039 households	Distance to water, type of water source, sanitation presence, ocular discharge, nasal discharge	TF/TI	Clinical exam	OR	Q	Q, O	O
Ngondi et al. [120]	Descriptive study in 25 villages in Southern Sudan	2005	1,712 children aged 1–9 years	Distance to water, sanitation presence, facial cleanliness, face-washing frequency, hygiene education	TF/TI	Clinical exam	OR	Q	Q, O	Q, O
Ngondi et al. [121]	Descriptive study in five trachoma hyperendemic districts of Amhara region	n.s.	1,813 randomly selected children aged 1–9 years from 912 households	Distance to water, sanitation presence, facial cleanliness, face-washing frequency	TF/TI	Clinical exam	OR	Q	Q, O	Q, O
Polack et al. [34]	Descriptive study in Shimbi Mashiriki village in northern Tanzania	2003	914 children aged 1–9 years for larger study, 233 children for sub-study of water allocation and trachoma	Distance to water, total quantity of water, quantity of water used for washing, sanitation maintenance, sanitation use, facial cleanliness, face-washing frequency, hand washing frequency	TF/TI, *C. trachomatis* infection	Clinical exam, PCR	OR	Q	Q	Q, O
Quicke et al. [122]	Descriptive study in the urban area of Brikama in The Gambia.	2012	652 children aged 1–9 years	Distance to water, type of water source, total quantity of water, sanitation presence, facial cleanliness, ocular discharge, nasal discharge, bathing frequency	TF	Clinical exam	OR	Q	Q	Q,O
Regassa et al. [123]	Descriptive study in Damot Gale District of South Ethiopia	2002	855 adults aged >15 years in randomly selected households	Distance to water, sanitation presence, face-washing frequency	TF/TI	Clinical exam	OR	Q	Q	Q
Schemann et al. [124]	Descriptive study in 30 villages in each of Mali's seven regions	1996–1997	Sample of 30 villages randomly selected in each of Mali's seven regions	Type of water source, sanitation presence, bathing frequency, face-washing frequency, facial cleanliness, flies on face, soap use, towel use	TF/TI, TI	Clinical exam	OR	O	O	Q, O
Schemann et al. [125]	Descriptive study in 9 villages in Segou and Mopti regions of Mali	2000–2002	Children aged <11 years	Distance to water, type of water source, sanitation presence, facial cleanliness, face-washing frequency, bathing frequency	TF/TI	Clinical exam	OR	Q	Q	Q, O
Taylor et al. [126]	Descriptive study in 20 villages in Kongwa district in central Tanzania	n.s.	Approximately 200 children aged 1–7 years from each of 20 villages	Distance to water, sanitation presence, facial cleanliness, towel use, nose wiping practices	TF/TI, TI	Clinical exam	OR	Q	Q, O	Q, O
Tielsch et al. [127]	Descriptive study in Lower Shire River Valley in Malawi	1983	5,436 children aged <6 years and 1,664 persons aged ≥6 years	Distance to water, sanitation presence, nose wiping practices, face-washing frequency	TI	Clinical exam	OR, 2×2	Q	Q	Q

H, hygiene; O, observed directly; Q, questionnaire; S, sanitation; W, water.

**Table 9:** Summary of meta-analyses examining association of WASH exposures with active trachoma (TF/TI) and *C. trachomatis* infection.

WASH Exposure	Active Trachoma (TF/TI)			*C. trachomatis* Infection		
	*n*	Random Effects Pooled OR (95% CI)	*I^2^* (95% CI)	*n*	Random Effects Pooled OR (95% CI)	*I^2^* (95% CI)
**Distance to water ≤1 km**	12	0.97 (0.83–1.11)	77 (60–87)	4	1.08 (0.86–1.30)	0 (0–85)
**Sanitation access**	26	0.85 (0.75–0.95)	75 (70–86)	7	0.67 (0.55–0.78)	0 (0–71)
**Sanitation use**	6	0.86 (0.57–1.15)	64 (12–85)	—	—	—
**Clean face**	25	0.42 (0.32–0.52)	78 (69–85)	—	—	—
**No ocular discharge**	10	0.42 (0.23–0.61)	68 (37–83)	4	0.40 (0.31–0.49)	0 (0–85)
**No nasal discharge**	9	0.62 (0.52–0.72)	30 (0–68)	4	0.56 (0.37–0.76)	8 (0–86)
**Washes face≥once daily**	6	0.76 (0.57–0.96)	70 (31–87)	—	—	—
**Washes face≥twice daily**	8	0.85 (0.80–0.90)	0 (0–68)	—	—	—
**Bathes≥once daily**	4	0.76 (0.53–0.99)	60 (0–87)	—	—	—
**Uses towel**	4	0.65 (0.53–0.78)	40 (0–80)	—	—	—
**Uses soap**	6	0.76 (0.59–0.93)	48 (0–79)	—	—	—

Seventy-six of the included publications were descriptive cross-sectional surveys, assessing at least one WASH-related condition or risk factor for *C. trachomatis* infection or clinical disease; ten publications involved some WASH-related intervention. Of the 453 effect measures from the included studies, 127 were reported as adjusted ORs, 269 were reported as unadjusted ORs, 20 were unadjusted ORs calculated from raw data using 2×2 tables, and 37 were reported as some other measure of effect (age-standardized rates, risk ratios, regression coefficients, etc.). Tansy Edwards, Joanne Katz, Jeremy Keenan, Caleb Mpyet, and Candace Vinke sent raw data in the form of 2×2 tables in response to a data request. Eighty percent of the studies were conducted in Africa, 10% were conducted in Asia, and 10% were conducted in Latin America. In general, the quality was low for studies included in the meta-analyses (see forest plots). These quality scores were due in part to the observational nature of nearly all studies, as well as the fact that WASH was the primary exposure of interest in only 32 of the studies.

### Definitions of WASH Characteristics

“Sanitation access” was generally assessed through interviews or questionnaires given to household head or child caretaker, sometimes verified through direct observation of the presence of a sanitation facility, which typically meant presence of a household-level, onsite, toilet or latrine facility. Access does not necessarily take into account use of the facility, which was generally assessed independently using questionnaires given to the head-of-household or a child's caregiver and, in some cases, verified through observation of indirect sanitation usage indicators (e.g., the presence of a beaten path to the door of the sanitation facility or the presence of feces). “Sanitation type” mainly referred to shared status or the location of a toilet or latrine (within house versus within compound). Each study assessing “facility maintenance” defined a different method of assessing and grading maintenance. All “sanitation education” was part of larger programs that also included a hygiene education component.

The time and distance increments used in examining the effect of “distance to water” varied from study to study, although the baseline for comparison was most commonly <30 min, or within 1 km to source. Because households often estimate distance to source in terms of travel time, WASH household surveys commonly equate a 30 min round trip from household to source (15 min each way) to a distance of 1 km (average walking speed of 4 km/hr) [31],[32]. As such, we standardized distance-to-source and time-to-source metrics where possible, according to this commonly used conversion, in order to include related ORs in a single meta-analysis. Similarly, there were no common volume increments used when evaluating the effect of “quantity of water used for washing” or “total quantity of water” collected by a household. The definition of “quantity of water used for washing” also varied by study (e.g., overall volume of water used for washing for the entire household versus volume used for individual children). “Water source type” most commonly examined the relative effects of community-specific water sources (e.g., tap versus draw-well versus hand-pump versus “other”, or indoor plumbing versus outdoor plumbing, comparing different types of wells, etc.) or compared “safe” and “unsafe” water sources as defined in each study. “Water access” as an exposure was poorly defined. In general, studies measured the presence of some specific water source within a village or the reported year-round availability of water.

The definition of “facial cleanliness” varied slightly from study to study, but generally was defined as the lack of ocular discharge, nasal discharge, and/or flies on face at the time of clinical examination. “Ocular discharge” included any discharge in or surrounding the eye, including “sleep” in eyes, and “nasal discharge” included any discharge present from the nose. “Face washing frequency” and “bathing frequency” were assessed using questionnaires and were most commonly reported as at least once daily (versus not daily) and at least twice daily (versus only daily or not daily) for face-washing frequency, and at least once daily (versus not daily) for bathing frequency. “Soap use” and “towel use” were also assessed using questionnaires and were reported as either use or no use. “Towel sharing” was defined as self-reported towel sharing or the availability of less than one towel per person. “Nose wiping practices” were also self-reported and compared children who wipe or blow nose using clothing or handkerchief and those who used neither. “Hygiene education” included both cross-sectional assessments of exposure or uptake of hygiene education and interventions involving hygiene education and promotion, including combined programs that also included sanitation education.

### Literature Density

**Figure 3:**
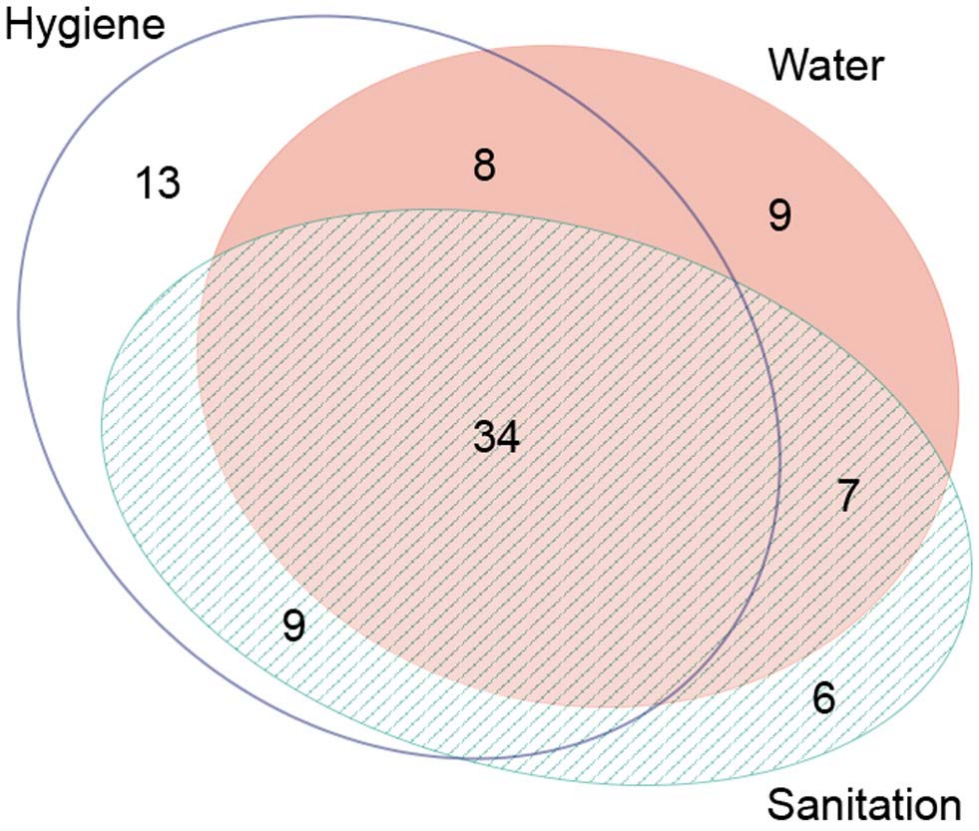
Publications reporting on the association between trachoma and water, sanitation, and hygiene exposures. Created using eulerAPE software.

Studies often examined more than one WASH exposure or condition and sometimes reported multiple effect measures (e.g., incremental measures of distance or face-washing frequency). Table 1 provides the total number of articles reporting on each WASH exposure, and Figure 3 shows the distribution of articles reporting on various combinations of WASH components. Effect measures, the number of parameters reported in the literature to compare a WASH exposure and trachoma outcome, are reported throughout the manuscript.

### Water-Related Exposures or Risk Factors

Fifty-eight of the studies identified in our systematic review reported at least one measure of effect for a water-related exposure or risk factor on trachoma, with a total 151 reported effect measures overall. Two intervention studies that improved access to water by installing water sources were identified; the remaining studies were observational. Study participants were chosen at random, either at individual or at household level in 69% of the relevant studies. In 14 studies, all individuals of a particular community, village, or special population group were enrolled, whereas no selection criteria for study participation were specified in four studies.

**Figure 4:**
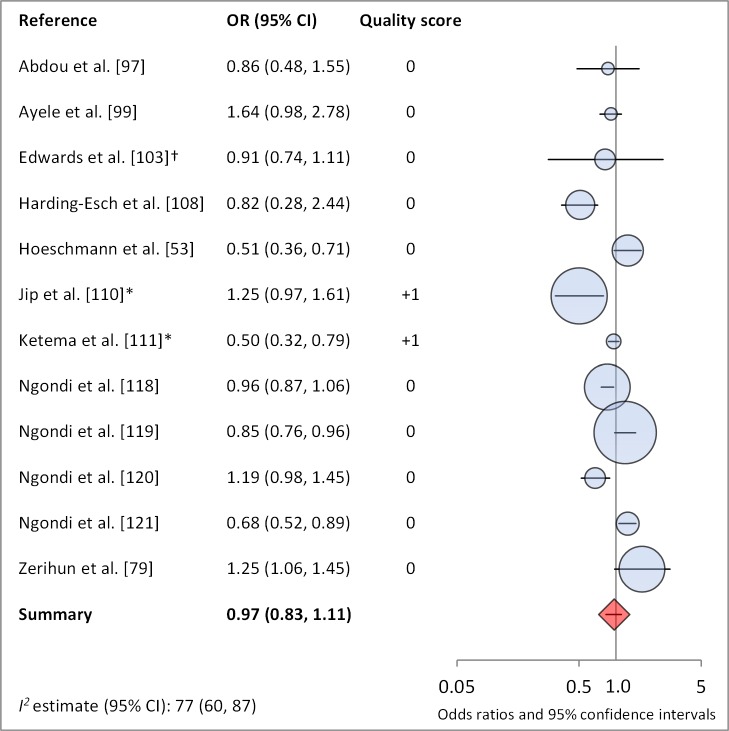
Meta-analysis examining the association of distance to water (≤1 km) with TF/TI. Circles indicate ORs, while the size of circles represents the sample size. Horizontal lines represent 95% confidence intervals. The diamond and corresponding line represent the random effects pooled OR and 95% confidence interval. *OR was adjusted for possible confounders. †OR was calculated using data sent from author.

**Figure 5:**
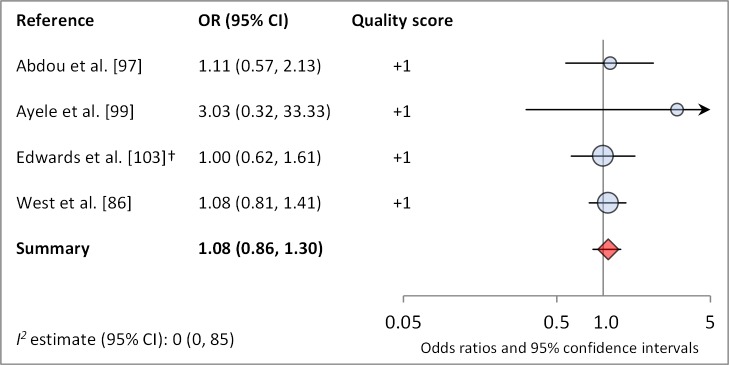
Meta-analysis examining the association of distance to water (≤1 km) with *C. trachomatis* infection. †OR was calculated using data sent from author.

Figures 4 and 5 illustrate the association between distance to water and TF/TI and *C. trachomatis* infection, respectively. Distance to water was reported in 38 publications, with a total reported 86 measures of effect. We included articles reporting on <1 km to water (or <30 min to collect water) in our meta-analyses [33]. We found no significant association between <1 km to water and TF/TI (OR 0.97, 95% CI 0.83–1.11) nor <1 km to water and *C. trachomatis* infection (OR 1.08, 95% CI 0.86–1.30).

Meta-analyses were not performed for other water-related conditions or risk factors owing to the lack of sufficient number of studies with comparable ORs and the variation in how exposures were measured and defined across studies (e.g., the type of water source reported in a study varied depending on context, and there were no common increments in measuring quantity of water collected or used for washing). The components for which meta-analyses were not conducted included: type of water source (20 articles, 37 effect measures), access to water (seven articles, seven effect measures), total quantity of water used for washing (six articles, 16 effect measures), and total quantity of water used daily within a household (six articles, nine effect measures). Despite the high number of water source-related studies identified, no meta-analyses were conducted because water source types tended to be poorly defined, and individual studies generally examined the relative effects of site-specific water sources, making comparison of across studies difficult. No clear significant effect of water source type was found across the articles, but, in general, those that showed a significant association between water source and trachoma found that “safer” water sources (those defined as “safe or protected” or piped water versus various types of wells versus an unprotected source) were protective against trachoma. “Access to water” was defined as either the presence of an improved water source or self-reported access to water year-round versus sporadically; most effect measures assessing access to water were not statistically significant (four effect measures), but those did find a significant effect suggest improved access is protective (three effect measures). Additionally, neither intervention that included the component improving water access led to a significant reduction in trachoma. Seven reported ORs suggest that using more water for washing is associated with significantly lower odds of trachoma, but the remaining nine reported ORs did not show a statistically significant relationship. Four of six studies reported a significant association between increased total quantity of water and lowered odds of trachoma. One study determined that, regardless of total water collection, using a higher proportion of the total household water for hygiene is protective [34].

### Sanitation-Related Exposures or Risk Factors

Fifty-six of the studies identified in our systematic review reported at least one measure of effect for a sanitation-related risk factor on trachoma, with a total of 96 reported effect measures overall. Four of these studies involved interventions to improve sanitation or sanitation/hygiene promotion; the remaining studies were observational. Study participants were chosen at random, either at individual or at household level in 68% of relevant studies. In 17 studies, all individuals of a particular community, village, or special population group were enrolled, whereas no selection criteria for study participation were specified in one study.

**Figure 6:**
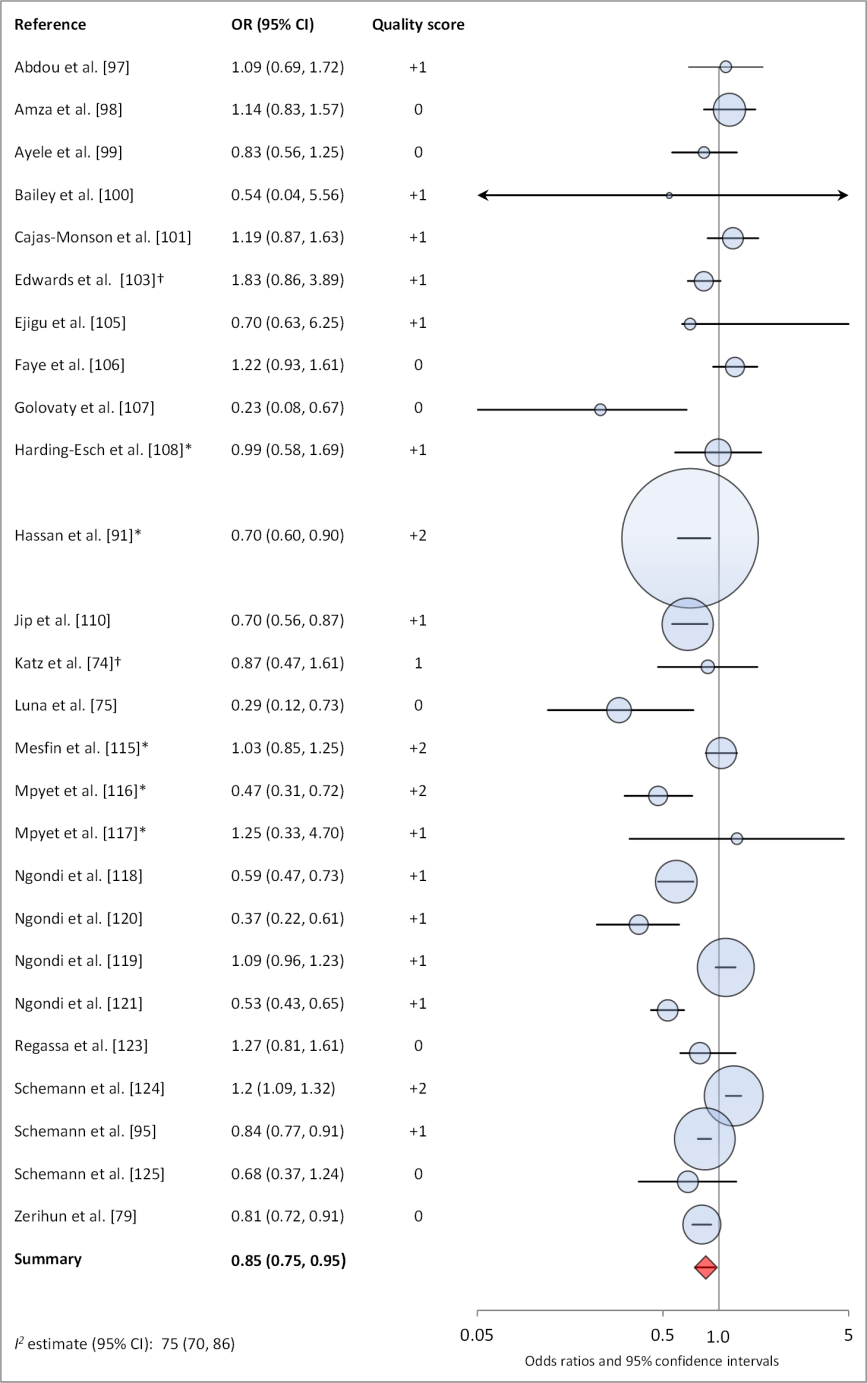
Meta-analysis examining the association of sanitation access with TF/TI. *OR was adjusted for possible confounders. †OR was calculated using data sent from author.

**Figure 7:**
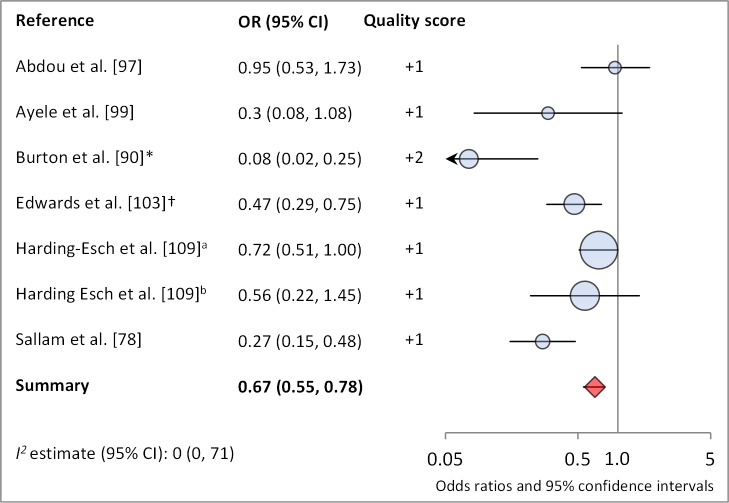
Meta-analysis examining the association of sanitation access with *C. trachomatis* infection. Results reported separately for ^a^The Gambia population, ^b^Tanzania population. *OR was adjusted for possible confounders. †OR was calculated using data sent from author.

**Figure 8:**
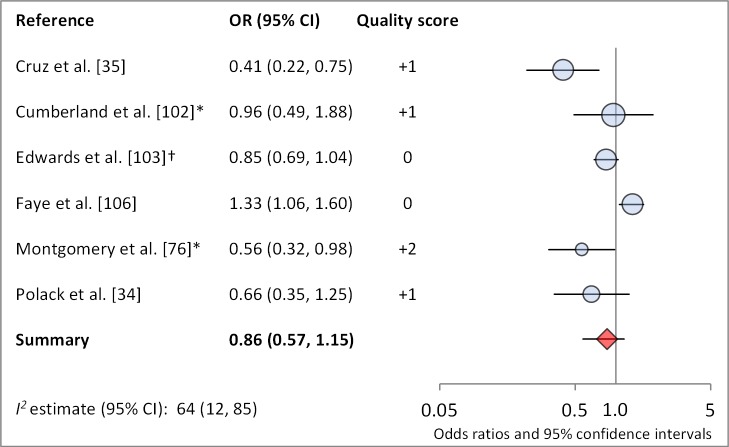
Meta-analysis examining the association of sanitation use with TF/TI. *OR was adjusted for possible confounders. †OR was calculated using data sent from author.

Figures 6 and 7 illustrate the association between sanitation and trachoma. We found that access to sanitation was associated with lower trachoma as measured by presence of TF/TI (OR 0.85, 95% CI 0.75–0.95) and *C. trachomatis* infection (OR 0.67, 95% CI 0.55–0.78). TF/TI was not significantly lower for those that reported higher levels of sanitation use (OR 0.86, CI 95% 0.57–1.15) (Figure 8).

Meta-analyses were not performed for other sanitation-related conditions or risk factors owing to a lack of sufficient number of studies with comparable ORs and the variation in how exposures were measured and defined across studies (e.g., there was no standard definition of either “facility maintenance” or “sanitation type”). These exposures included sanitation type (six articles, 11 effect measures), sanitation education (three articles, six effect measures), and facility maintenance (three articles, five effect measures). One study found the presence of a sanitation facility within the house to be significantly protective against TF/TI compared to facilities located outside of the house [35], and another found that a facility with no privacy may be associated with increased odds of TF [36]. The remaining four studies found no association between type of sanitation and trachoma. Three studies examined the effect of sanitation education or promotion on trachoma, but these analyses were part of larger programs also addressing hygiene and face washing, thus sanitation education could not be isolated. Two of three studies showed FE combined education intervention villages had significantly lower trachoma at follow-up. One study examined the effect of sanitation promotion without hygiene education but found no statistically significant effect [37].

### Hygiene-Related Exposures or Risk Factors

Sixty-four studies identified in our systematic review reported some association between a hygiene-related exposure and trachoma, with a total of 213 reported measures of effect. Six studies assessed the effect of a hygiene education intervention on trachoma; all other studies were observational. Study participants were chosen at random, either at individual or at household level in 61% of relevant studies. In 23 studies, all individuals of a particular community, village, or special population group were enrolled, whereas no selection criteria for study participation were specified in two studies.

**Figure 9:**
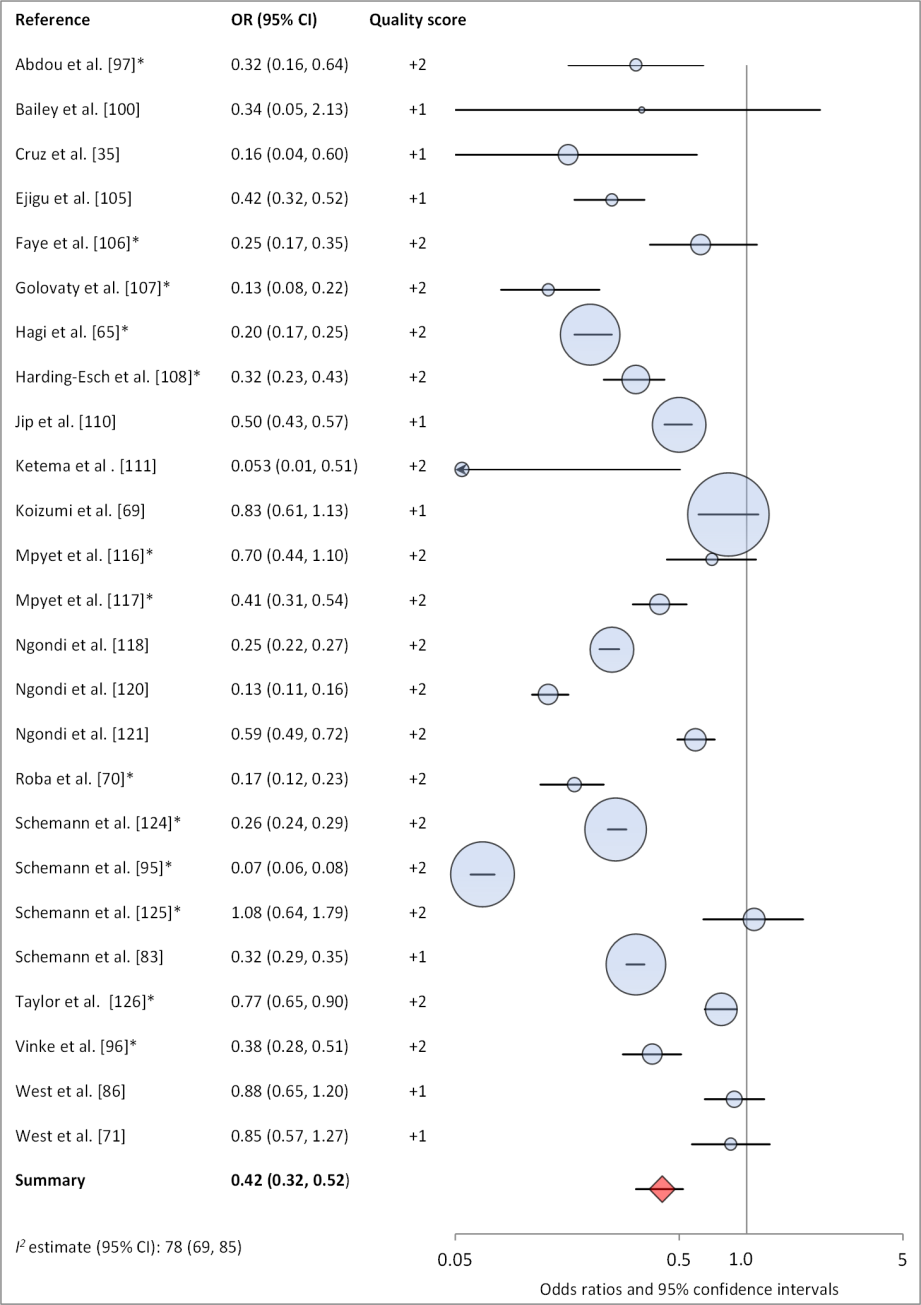
Meta-analysis examining the association of clean face with TF/TI. *OR was adjusted for possible confounders.

**Figure 10:**
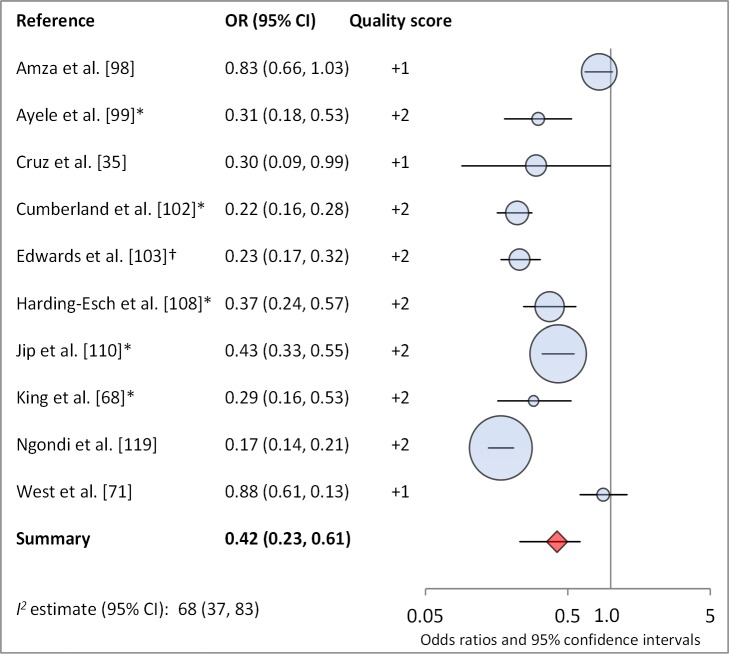
Meta-analysis examining the association of no ocular discharge with TF/TI. *OR was adjusted for possible confounders. †OR was calculated using data sent from author.

**Figure 11:**
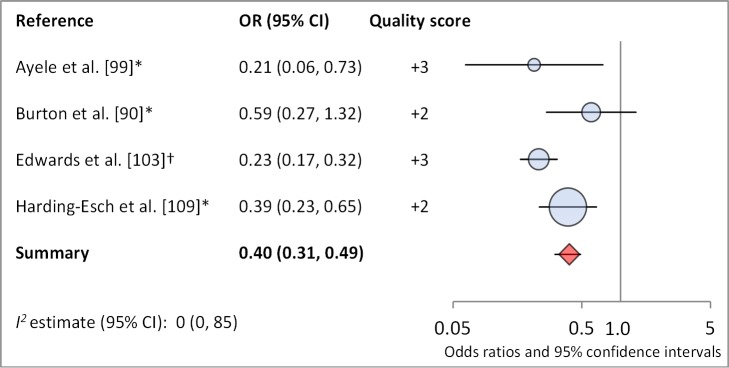
Meta-analysis examining the association of no ocular discharge with *C. trachomatis* infection. *OR was adjusted for possible confounders. †OR was calculated using data sent from author.

**Figure 12:**
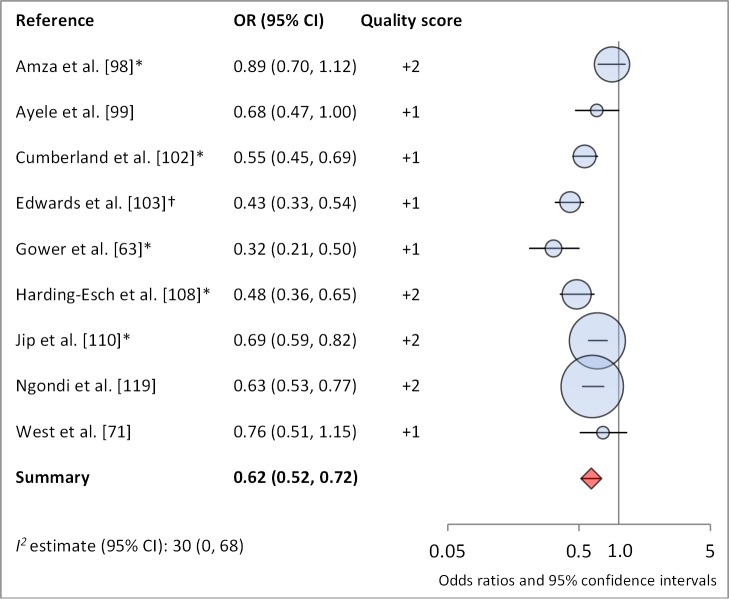
Meta-analysis examining the association of no nasal discharge with TF/TI. *OR was adjusted for possible confounders. †OR was calculated using data sent from author.

**Figure 13:**
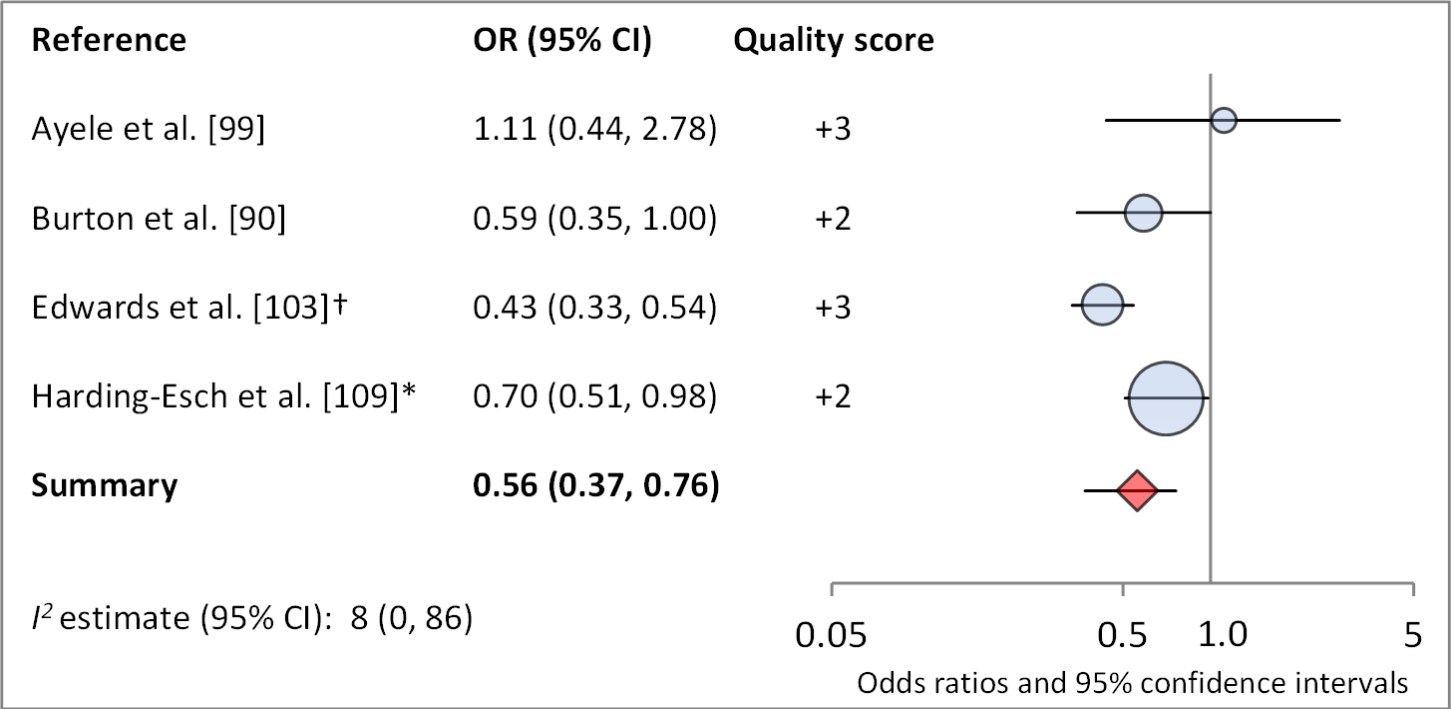
Meta-analysis examining the association of no nasal discharge with *C. trachomatis* infection. *OR was adjusted for possible confounders. †OR was calculated using data sent from author.

**Figure 14:**
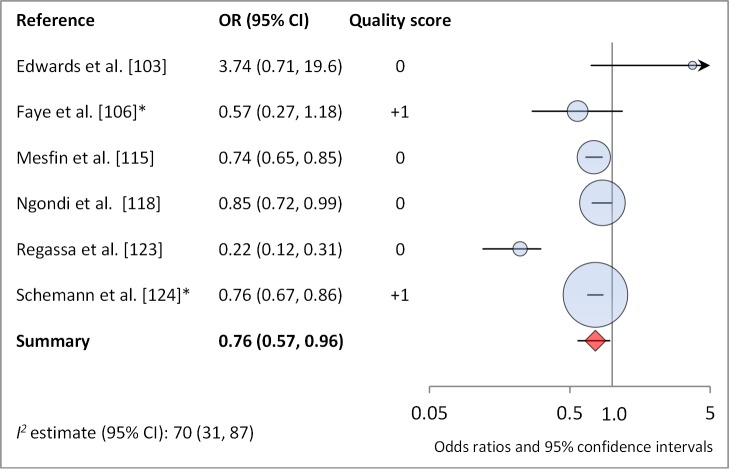
Meta-analysis examining the association of washing face ≥1 time per day (versus <1 time per day) with TF/TI. *OR was adjusted for possible confounders.

**Figure 15:**
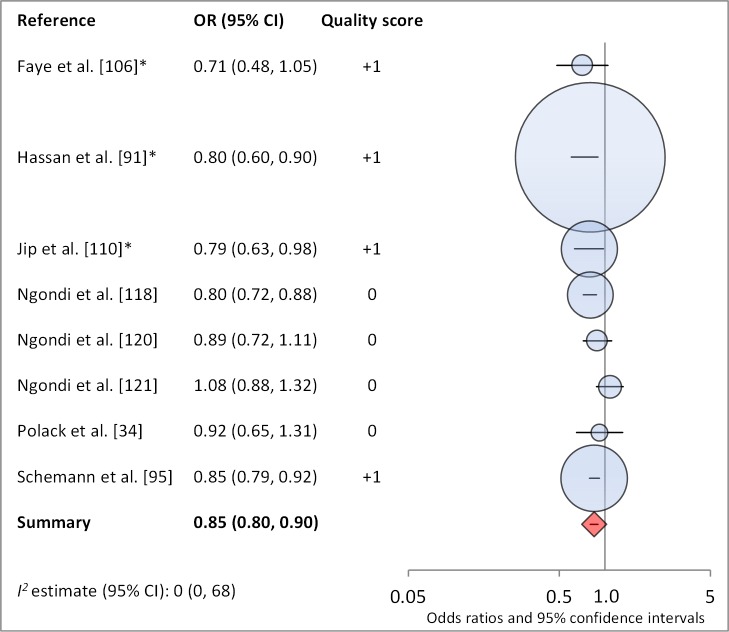
Meta-analysis examining the association of washing face ≥2 times per day (versus <2× per day) with TF/TI. *OR was adjusted for possible confounders.

**Figure 16:**
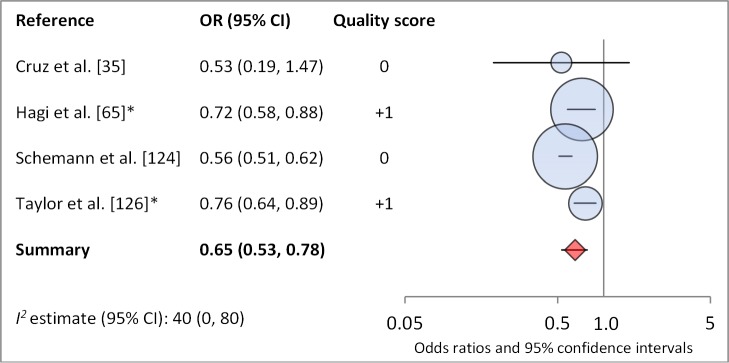
Meta-analysis examining the association of towel use with TF/TI. *OR was adjusted for possible confounders.

**Figure 17:**
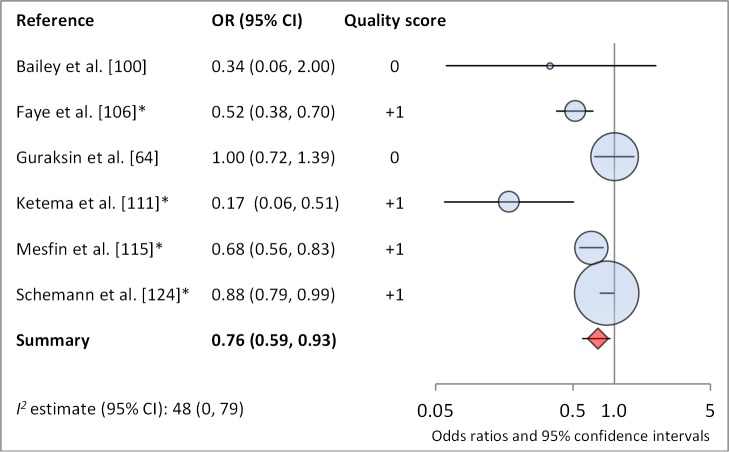
Meta-analysis examining the association of soap use with TF/TI. *OR was adjusted for possible confounders.

**Figure 18:**
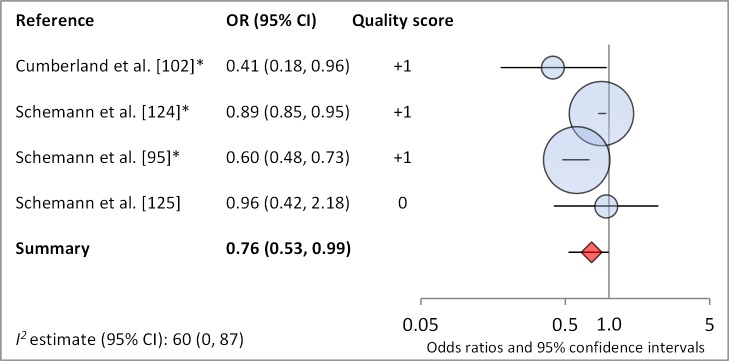
Meta-analysis examining the association of bathing at least once daily with TF/TI. *OR was adjusted for possible confounders.

Facial cleanliness status was reported in 35 publications, with a total reported 49 measures of effect. We found that having a clean face was protective against TF/TI (OR 0.42, 95% CI 0.32–0.52) (Figure 9). No ocular discharge at the time of examination was associated with reduced odds of both TF/TI (OR 0.42, 95% CI 0.23–0.61) (Figure 10) and *C. trachomatis* infection (OR 0.40, 95% CI 0.31–0.49) (Figure 11). We also found significant associations between lack of nasal discharge and reduced odds of both TF/TI (OR 0.62, 95% CI 0.52–0.72) (Figure 12) and *C. trachomatis* infection (OR 0.56, 95% CI 0.37–0.76) (Figure 13). The relationship between TF/TI and face washing at least once a day (OR 0.76, 95% CI 0.57–0.96) (Figure 14) was stronger than the relationship between TF/TI and washing at least twice a day (OR 0.85, 95% CI 0.80–0.90) (Figure 15), and soap use was significantly associated with reduced TF/TI (OR 0.76, 95% CI 0.59–0.93) (Figure 16). Few studies reported measures of effect between TF/TI and bathing at least once a day (OR 0.76, 95% CI 0.53–0.99) (Figure 17) or towel use (OR 0.65, 95% CI 0.53–0.78) (Figure 18).

Meta-analyses were not performed for other hygiene-related conditions or risk factors due to a lack of sufficient number of studies with comparable ORs, often resulting from variation in how exposures were measured and defined across studies (e.g., the nose-wiping practices of interest varied from study to study, hygiene education interventions may or may not have included a sanitation component). These exposures included towel sharing (three articles, four measures of effect), hygiene education (six articles and 12 measures of effect), and nose-wiping practices (five articles, seven measures of effect). Towel sharing among family members was not found to be significantly associated with trachoma in any study. One of two interventions focused solely on face-washing education reported a significant reduction in severe trachoma (TI) compared to control villages [38]. Two of the three interventions that combined hygiene and sanitation education components showed a significant reduction in TF/TI in villages receiving education. All five studies addressing nose-wiping practices reported significant effects: three studies showed that using clothing to blow nose is a significant risk factor for some form of clinical trachoma; the remaining two studies showed significant associations between handkerchief use and lower odds of TF/TI, TI, and *C. trachomatis* infection.

## Discussion

Our analysis revealed evidence of an association between improved WASH conditions and exposures and reduced trachoma in 11 of the 15 meta-analyses conducted with the available literature. Pooled estimates of effect for the relationship between a WASH exposure and clinical trachoma (TF/TI) ranged from strong effects associated with a clean face (OR 0.42) to little evidence of an association for distance to water source <1 km (OR 0.97). Three of the four meta-analyses assessing trachoma through PCR found associations between WASH conditions and lower infection with *C. trachomatis*. Access to sanitation was strongly associated with lower levels of trachoma, both TF/TI and *C. trachomatis*. The relative strength of the association between sanitation access and *C. trachomatis* infection compared to TF/TI may be due in part to the persistence of clinical signs of trachoma for some time after infection is eliminated [39]. Only seven studies reported estimates of the relationship between sanitation use and TF/TI, and while the meta-analysis suggested an association that was not statistically significant.

We found the strongest evidence of the association between hygiene factors and trachoma. Our analyses suggest that the presence of a clean face, the lack of ocular and nasal discharge, increased frequency of face washing, towel use, the use of soap, and daily bathing were all associated with lower odds of trachoma. The relationship between a clean face and reduced odds of trachoma was one of the strongest associations. The shared use of towels, which has long been thought to be a key risk factor in the transmission of trachoma [40]–[42], was not found to be significantly associated with increased risk of trachoma in any of the identified studies, though too few studies were available to conduct a meta-analysis. Our meta-analysis suggest that facial cleanliness and the absence of either ocular or nasal discharge are highly associated with decreased odds of infection with *C. trachomatis*. However, there is a potentially tautological effect, as trachoma causes inflammation of the eyes, which results in lacrimation and ocular discharge that contains contagion [43]. It stands to reason that removing ocular discharge removes contagion, but ocular discharge itself may also be a symptom of trachoma, and so ocular discharge and active trachoma are both risk factors and effects. However, considering the strong association between facial cleanliness and nasal discharge and trachoma infection, our results suggest that health behaviors that result in clean faces may reduce the prevalence of trachoma. Our meta-analysis examining daily face-washing practices suggests that washing once a day may decrease odds of TF/TI, although washing more than once a day does not appear to result in an additional decrease. The use of soap was associated with lower levels of trachoma, which is particularly relevant as current strategies for trachoma control advocate for face washing with water, but do not consistently emphasize the use of soap [41].

Our analysis did not reveal any evidence of an assocation between the distance to drinking water source and *C. trachomatis infection* or TF/TI. The lack of an observed effect may be due in part to the 1 km cutpoint used in most studies, which may not be a meaningful standard with respect to trachoma control. A more proximate drinking water source may serve as an indicator for increased quantity of water [32], but is not a guarantee of improved water quality [44]. Given the strong association found between hygiene and trachoma, a lower cutoff value for distance to water source, which ensures more assuredly adequate water quantity for household uses, may be preferable. We found a number of studies that reported an association between improved water quantity and reduced odds of trachoma, but there was an insufficient number of articles to conduct a meta-analysis. Data on water access were limited by lack of standardization of definitions and metrics and the resulting low comparability across studies.

Our review identified relationships between WASH conditions and trachoma that require additional research. While we found several studies assessing the association of household sanitation access and facial cleanliness on signs of trachoma, there are comparatively fewer exploring the relationship between water use and trachoma, as well as other characteristics of sanitation, such as maintenance of sanitation facilities and use of facilities shared by more than one household. As a result, the association of these variables with trachoma and the potential role of these variables in trachoma control remains unknown.

### Strengths and Limitations

We searched three widely used databases—EMBASE, Web of Science, and PubMed—as well as MedCarib, Lilacs, REPIDISCA, DESASTRES, and African Index Medicus using a pre-specified, systematic search protocol. In addition, we hand-searched the bibliographies of available reviews. We adhered to the MOOSE guidelines for reporting meta-analysis of observational studies. The lack of standard definitions and methods of measuring WASH components in the available studies made meta-analysis for certain exposures infeasible. In addition, variations in the definitions and methods could have introduced error in our meta-analyses. Study design and setting varied, and heterogeneity was moderate to high in some meta-analyses, particularly those examining the relationship between WASH and TF/TI (refer to *I^2^* values and 95% confidence intervals in Table 9). Although infection with *C. trachomatis* is a more objective measure of trachoma and is likely associated with more temporal access to WASH, it was the outcome of interest in only four of our 15 meta-analyses, as most studies reported only on clinically evident signs of TF/TI, which could be the result of past infection and more related to cumulative exposure to risk factors. Overall, funnel plots did not appear to show high publication bias (Figure S1). However, it is clear from our review that the majority of studies that report on trachoma prevalence do not report on associations with WASH. Many studies only reported the statistically significant ORs from multivariable analysis. In these cases, ORs available from the univariable were used in meta-analyses; however, as these univariable ORs are not adjusted for possible confounders, they may not be as reliable as ORs from multivariable analyses. It is difficult to draw solid conclusions for those WASH exposures for which meta-analyses were not possible due to scarcity of literature (e.g., facility maintenance and quality, water source type), and we could be missing important insights into the relationship between trachoma and other aspects of WASH. Finally, there is a lack of independence between our meta-analyses estimates, since many studies report multiple measures of effect that contribute to several analyses.

All studies included in meta-analyses had relatively low quality scores. These low-quality grades were due in part to the fact that only observational studies were included in the meta-analyses, and all ORs reflected cross-sectional data. WASH exposures were often assessed as secondary risk factors of interest (e.g., sanitation access could be included in a model focused on assessing an antibiotic intervention), meaning that there is a potential for publication bias if non-significant associations were not included in final published manuscripts.

We found many different measures of WASH exposures and many of these parameters were poorly described in the methods. This lack of specificity and standardization of measures limits the harmonization of findings into a meta-analysis; this creates challenges to amassing a consistent and useable body of evidence, and effectively demonstrating impact across the WASH and disease control sectors. In addition, we only reported measures of TF/TI together. Since TI has been dropped from some surveys in recent years, our measures of effect may represent small differences in the definition of the outcome.

### Policy Implications

While there has been considerable progress in global access to improved drinking water supply, specifically in East and South Asia, there is evidence that these gains have systematically ignored the poorest and most marginalized populations [45],[46], which are precisely the most vulnerable to trachoma. In addition, the world is far from achieving the Millennium Development Goal target of reducing by half the global population without access to sanitation. In sub-Saharan Africa, which has the world's highest burden of trachoma, fewer than 50% of households have access to an improved sanitation facility, and more than 25% of households practice open defecation [47]. If elimination of trachoma is to be achieved, additional resources must be targeted towards sustainable access to WASH in trachoma endemic areas. Recent discourse between the neglected tropical disease (NTD) control programs and those in the WASH sector may lead to more tangible efforts at collaboration, coordination, and cooperation between the sectors [17], which are essential to achieving articulated WASH strategies, and targeted WASH implementation for trachoma reduction [12].

Face washing has long been an elemental component of the SAFE strategy. Among NGOs not specifically targeting trachoma control, however, improving WASH access is typically promoted to reduce diarrheal diseases, and as such few promote face washing alongside handwashing with soap. Our data support the importance, found by others, of daily face washing to reduce trachoma. Increasing attention to and investment in improved hygiene practices that include daily face washing will support the achievement of trachoma control and elimination targets.

Access to household sanitary facility is an important factor in trachoma, and continued use of program indicators that record progress toward improving household access to sanitation is recommended. Though sanitation use, in addition to access, is theoretically important to control trachoma, our review found no significant association between measures of sanitation use and trachoma. This may suggest that current measures of sanitation use, which vary between observation of sanitation characteristics and direct household surveys related to defecation practices, are not sufficiently accurate or consistent on the whole. Additionally, despite a scarcity of literature examining the role of sanitation facility quality or maintenance, interventions to improve the usability and acceptability of existing sanitation facilities may be effective. Further research is needed to understand whether sanitation access at a household level, or whether reaching high sanitation coverage within the community as a whole is more important to achieving trachoma reduction. None of these studies focused on schools, revealing a gap in the literature of the importance of school-based WASH promotion.

### Conclusions

Our review finds that the F and E components of the SAFE strategy—specifically face washing, facial cleanliness, and sanitation access—but also general hygiene behaviors for trachoma control are well supported by evidence. Facial cleanliness and environmental improvement are, in general, highly associated with trachoma infection and disease. Lack of evidence of the association between water access and trachoma may be due to the low number of studies available or a parameterization of water access (<1 km distance to source) in a majority of studies that does not best capture the impact of increased access to water for hygiene and cleanliness. These data support the need for a more harmonized approach to monitoring WASH exposures as they relate to trachoma, better monitoring and reporting of these associations, and increased attention to and funds for the use of WASH to meet trachoma elimination targets as part of the full SAFE strategy. Further studies should employ WASH definitions compatible with those used in the WASH sector, such as the Joint Monitoring Program definitions for improved and unimproved water sources and infrastructure [47]. There is, furthermore, a clear need for more robust evidence of WASH impact on trachoma that may be achieved by including baseline and follow-up measurements of trachoma in impact evaluations of WASH programs and activities.

## Supporting Information

Figure S1 **Funnel plots for publication bias.**
(DOCX)

Text S1 **PRISMA checklist.**
(DOC)

Text S2 **Study protocol.**
(DOC)

Text S3 **Potential confounders controlled for in individual studies.**
(XLSX)

Text S4 **Study quality grade by meta-analysis.**
(XLSX)

## Abbreviations

OR: odds ratio
TF: trachomatous inflammation-follicular
TI: trachomatous inflammation-intense
WASH: water, sanitation, and hygiene
