# Supplementary material for: Effect of Water, Sanitation, and Hygiene on the Prevention of Trachoma: A Systematic Review and Meta-Analysis
Source: PLoS Med. 2014 Feb 25;11(2):e1001605. doi: 10.1371/journal.pmed.1001605 (PMC3934994; doi:10.1371/journal.pmed.1001605)
Supplement: Figure S1 — Funnel plots for publication bias. (DOCX) [file pmed.1001605.s001.docx]

**Figure S1. Distance to water ≤ 1 km and TF/TI.**

**Figure S2. Distance to water ≤ 1 km and *C. trachomatis* infection.**

**Figure S3. Latrine access and TF/TI.**
 **Figure S4. Latrine access and *C. trachomatis* infection.**

**Figure S5. Latrine use and TF/TI.**

**Figure S6. Washes face daily and TF/TI.**

**Figure S7. Washes face at least twice daily and TF/TI.**

**Figure S8. Facial Cleanliness and TF/TI.**

**Figure S9. No ocular discharge and TF/TI.**

**Figure S10. No ocular discharge and *C. trachomatis* infection.**

**Figure S11. No nasal discharge and TF/TI.**

**Figure S12. No nasal discharge and *C. trachomatis* infection.**

**Figure S13. Towel use and TF/TI.**

**Figure S14. Soap use and TF/TI.**

**Figure S15. Bathes daily and TF/TI.**
