# Supplementary material for: Effect of Water, Sanitation, and Hygiene on the Prevention of Trachoma: A Systematic Review and Meta-Analysis
Source: PLoS Med. 2014 Feb 25;11(2):e1001605. doi: 10.1371/journal.pmed.1001605 (PMC3934994; doi:10.1371/journal.pmed.1001605)
Supplement: Text S2 — Study protocol. (DOC) [file pmed.1001605.s003.doc]

**Text S2 Study protocol for systematic review and meta-analyses to evaluate the effect of water, sanitation, and hygiene on trachoma infection and disease**

**Objective**

To examine the effect of water, sanitation, and hygiene (WASH) conditions and risk factors on trachoma disease and infection with *Chlamydia trachomatis*.

**Reporting**

We will adhere to the PRISMA (Preferred Reporting Items for Systematic Reviews and Meta-Analyses) statement and the MOOSE (Meta-analysis of Observational Studies in Epidemiology) guidelines for reporting results.

**Search Methods**

We will systematically search Pubmed, Embase, and Web of Science for relevant articles. We will also index relevant studies from the bibliographies of seven related review papers. We will not restrict results based on language or publication date. Our search will be performed until May 14, 2013.

Our database search will include the term [trachom*] in combination with the following series of WASH-related keywords: [clean* fac*], [environment*], [excre*], [face washing], [facewashing], [faec*], [fec*], [hand washing], [handwashing], [hygiene], [latrine*], [sanitation], [toilet*], [towel*], [wash cloth*], [waste], and [water].

Note: An asterisk (*) denotes a wildcard character.

**Eligibility**

*Inclusion criteria*

Article eligibility will be determined by three key criteria. Studies must contain:

1. A distinctly identifiable measurement of WASH variable(s)
2. A direct measurement of trachoma (e.g. *C. trachomatis* infection, clinically diagnosed disease)
3. A distinct parameterization of WASH on trachoma (e.g. odds ratio)

*Study type and participants*

All study types will be eligible for inclusion. There will be no restrictions on setting or study population (e.g. certain age groups or other high-risk populations).

*WASH-related conditions and risk factors*

The definition of water, sanitation, and hygiene practices or conditions will be broad, but will not include exclusively vector-focused factors, such as fly-eye contact, presence of flies in home, or insecticide-based interventions. Data collected through direct observation and through questionnaires/interviews will be eligible for inclusion.

*Outcome measures*

The reported odds ratios (ORs) describing the effect of WASH factors on trachoma will serve as effect measures. We will collect both crude and adjusted estimates where available. If ORs are not reported, they will be calculated from data provided in articles; if insufficient data is provided within the text, authors will be contacted for additional information (e.g. raw data provided in 2x2 tables). For the purposes of comparison, ORs will be standardized so that an OR less than 1.0 indicates a decrease in the odds of trachoma among those with *improved* WASH conditions (e.g. more frequent face washing, having access to a latrine, living within 1 km of a water source).

WASH risk factor meta-analyses will be conducted when three or more studies are identified reporting comparable ORs (e.g. three or more ORs describing the effect of living within 1 km of a water source on TF/TI, or three or more ORs describing the effect of using a latrine on being diagnosed with *C. trachomatis* infection). When adjusted ORs are available, they will be used in meta-analysis; when no adjusted estimates are available, unadjusted ORs will be used. For those WASH risk factors where meta-analyses are not possible, ORs and overall trends will be summarized briefly within the text.

**Data Collection**

Two reviewers (MS, CM) will independently examine titles and abstracts to determine the relevance of each article. Reviewers will base final selection on the full text of potentially relevant articles. A third reviewer (MF) will be consulted to determine eligibility of articles in cases of disagreement.

Relevant data from all eligible studies will be collected by one reviewer (MS) into a designated spreadsheet. Extracted data will include:

- Study description (e.g. timeframe, study design, setting, sample size)
- Characteristics of the study population (e.g. age group, socioeconomic details)
- The selection process (e.g. random selection)
- Details about WASH factors and how data was collected (e.g. water access observed by research team, latrine use assessed through questionnaires)
- Details on diagnosis of trachoma (e.g. *C. trachomatis* infection diagnosed using PCR, TF/TI diagnosed during clinical examination of eyelids)
- Any other relevant information (e.g. specific strengths or limitations mentioned by authors)

**Assessing quality**

The research team will develop quality grading criteria based on the GRADE methodology . Because GRADE is not primarily intended for observational studies, we will base our key bias categories on those used for a similar review on soil-transmitted helminthes and modify them as necessary to apply to trachoma-focused studies.

Using the score system outlined in Table 1, MS will assign a grade to each study for every WASH-related factor that is included in a meta-analysis (e.g. one study may have one grade specific to latrine access and another grade specific to latrine use, as points related to exposure assessment will vary by WASH exposure). No study will be excluded due to quality score.

| **Table 1: Measures & Descriptions for Bias Assessment Score** | **Points** |
| --- | --- |
| *Diagnostics* | |
| Rigorous diagnostic approach (e.g. PCR or other laboratory technique used to assess *c. trachomatis* infection status) | +1 |
| No laboratory-based diagnostic approach (e.g. clinical examination only) | 0 |
| *Exposure Assessment* | |
| Exposure was observed directly by the research team (e.g. ocular discharge noted at time of examination) | +1 |
| Exposure was assessed using a questionnaire (e.g. self-reported face washing) | 0 |
| *Study Design* | |
| Observational study | 0 |
| Quasi-experimental design (e.g. matched control, before and after) | +1 |
| Experimental design (RCT or CRT = inclusion of randomized control ) | +2 |
| Purposively calculated sample size to address trachoma as a WASH outcome | +1 |
| *Other Strengths and Limitations* | |
| Attempted to control for confounding variables | +1 |
| Other strengths | +1 |
| Other limitations | -1 |

**Meta-analysis**

We will conduct meta-analyses for individual WASH factors when sufficient data is available (i.e. three or more studies reporting ORs describing the relationship between a similar WASH factor and the same disease outcome). Subcomponent analyses will be divided into *C. trachomatis* infection and clinically diagnosed active trachoma (TF/TI).

We will assess publication bias through qualitative funnel plot analyses, and Moran’s I^2^ and Cochrane’s Q-tests will be utilized to evaluate heterogeneity. When heterogeneity is high (I^2^>50%), we will use random effects models for meta-analyses . Our dependent variable will be the natural log of the reported odds ratios, and studies will be weighted based on their standard errors.

**References**

Atkins, D., D. Best, P. A. Briss, M. Eccles, Y. Falck-Ytter, S. Flottorp, G. H. Guyatt, R. T. Harbour, M. C. Haugh, D. Henry, S. Hill, R. Jaeschke, G. Leng, A. Liberati, N. Magrini, J. Mason, P. Middleton, J. Mrukowicz, D. O'Connell, A. D. Oxman, B. Phillips, H. J. Schunemann, T. Edejer, H. Varonen, G. E. Vist, J. W. Williams, Jr. and S. Zaza (2004). "Grading quality of evidence and strength of recommendations." BMJ **328**(7454): 1490.

DerSimonian, R. and N. Laird (1986). "Meta-analysis in clinical trials." Control Clin Trials **7**(3): 177-188.

Stroup, D. F., J. A. Berlin, S. C. Morton, I. Olkin, G. D. Williamson, D. Rennie, D. Moher, B. J. Becker, T. A. Sipe and S. B. Thacker (2000). "Meta-analysis of observational studies in epidemiology: a proposal for reporting. Meta-analysis Of Observational Studies in Epidemiology (MOOSE) group." JAMA **283**(15): 2008-2012.

Ziegelbauer, K., B. Speich, D. Mausezahl, R. Bos, J. Keiser and J. Utzinger (2012). "Effect of sanitation on soil-transmitted helminth infection: systematic review and meta-analysis." PLoS Med **9**(1): e1001162.
